# Multibridge: an R package to evaluate informed hypotheses in binomial and multinomial models

**DOI:** 10.3758/s13428-022-02020-1

**Published:** 2023-06-05

**Authors:** Alexandra Sarafoglou, Frederik Aust, Maarten Marsman, František Bartoš, Eric-Jan Wagenmakers, Julia M. Haaf

**Affiliations:** Department of Psychology, University of Amsterdam, PO Box 15906 1001 NK Amsterdam, The Netherlands

**Keywords:** Bayes factors, Model selection, Inequality constraints, Savage-Dickey density ratio, Bridge sampling

## Abstract

The **multibridge** R package allows a Bayesian evaluation of informed hypotheses ${\mathscr{H}}_{r}$ applied to frequency data from an independent binomial or multinomial distribution. **multibridge** uses bridge sampling to efficiently compute Bayes factors for the following hypotheses concerning the latent category proportions ***𝜃***: (a) hypotheses that postulate equality constraints (e.g., *𝜃*_1_ = *𝜃*_2_ = *𝜃*_3_); (b) hypotheses that postulate inequality constraints (e.g., *𝜃*_1_ < *𝜃*_2_ < *𝜃*_3_ or *𝜃*_1_ > *𝜃*_2_ > *𝜃*_3_); (c) hypotheses that postulate combinations of inequality constraints and equality constraints (e.g., *𝜃*_1_ < *𝜃*_2_ = *𝜃*_3_); and (d) hypotheses that postulate combinations of (a)–(c) (e.g., *𝜃*_1_ < (*𝜃*_2_ = *𝜃*_3_),*𝜃*_4_). Any informed hypothesis ${\mathscr{H}}_r$ may be compared against the encompassing hypothesis ${\mathscr{H}}_e$ that all category proportions vary freely, or against the null hypothesis ${\mathscr{H}}_{0}$ that all category proportions are equal. **multibridge** facilitates the fast and accurate comparison of large models with many constraints and models for which relatively little posterior mass falls in the restricted parameter space. This paper describes the underlying methodology and illustrates the use of **multibridge** through fully reproducible examples.

## Introduction

The most common way to analyze categorical variables is to conduct either binomial tests, multinomial tests, or chi-square goodness of fit tests. These tests compare the encompassing hypothesis to a null hypothesis that all underlying category proportions are either exactly equal, or follow a specific distribution. Accordingly, these tests are suitable when theories predict either the invariance of all category proportions or specific values. For instance, chi-square goodness of fit tests are commonly used to test Benford’s law, which predicts the distribution of leading digits in empirical datasets (Benford, 1938; Newcomb, 1881). Often, however, the predictions that researchers are interested in are of a different kind. Consider for instance the weak-order mixture model of decision-making (Regenwetter & Davis-Stober, 2012). The theory predicts that individuals’ choice preferences are weakly ordered at all times, that is, if they prefer choice *A* over *B* and *B* over *C* then they will also prefer *A* over *C* (Regenwetter, Dana, & Davis-Stober, 2011)—a well-constrained prediction of behavior. The theory is, however, silent about the exact values of each choice preference. Hence, the standard tests that compare ${\mathscr{H}}_{e}$ to ${\mathscr{H}}_{0}$ are unsuited to test the derived predictions. Instead, the predictions need to be translated into an informed hypothesis ${\mathscr{H}}_{r}$ that reflects the predicted ordinal relations among the parameters. Only then is it possible to adequately test whether the theory of weakly-ordered preference describes participants’ choice behavior. Of course, researchers may be interested in more complex hypotheses, including ones that feature combinations of equality constraints, inequality constraints, and unconstrained category proportions. For instance, Nuijten, Hartgerink, Assen, Epskamp, and Wicherts (2016) hypothesized that articles published in social psychology journals would have higher error rates than articles published in other psychology journals. As in the previous example, the authors had no expectations about the exact error rate distribution across journals. Here, again, the standard tests are inadequate. Generally, by specifying informed hypotheses researchers and practitioners are able to “add theoretical expectations to the traditional alternative hypothesis” (Hoijtink et al., 2008, p. 2) and thus test hypotheses that relate more closely to their theories (Haaf, Klaassen, & Rouder, 2019; Rijkeboer & Van Den Hout, 2008).

In the Bayesian framework, researchers may test hypotheses of interest by means of Bayes factors (Jeffreys, 1935; Kass & Raftery, 1995). Bayes factors quantify the extent to which the data change the prior model odds to the posterior model odds, that is, the extent to which one hypothesis outpredicts the other. Specifically, Bayes factors are the ratio of marginal likelihoods of the respective hypotheses. For instance, the Bayes factor for the informed hypothesis versus the encompassing hypothesis is defined as:

$$ \begin{array}{@{}rcl@{}} \text{BF}_{re} = \frac{\overbrace{p(\mathbf{x}\mid \mathcal{H}_{r})}^{\underset{\text{\small under} \mathcal{H}_{r}}{\text{\small Marginal likelihood}}}}{\underbrace{p(\mathbf{x}\mid \mathcal{H}_{e})}_{\underset{\text{\small under} \mathcal{H}_{e}}{\text{\small Marginal likelihood}}}}, \end{array} $$

where the subscript *r* denotes the informed hypothesis and *e* denotes the encompassing hypothesis. Several available R packages compute Bayes factors for informed hypotheses. For instance, the package **multinomineq** (Heck & Davis-Stober, 2019) evaluates informed hypotheses for multinomial models as well as models that feature independent binomials. The package **BFpack** (Mulder et al., 2021) evaluates informed hypotheses for statistical models such as univariate and multivariate normal linear models, generalized linear models, special cases of linear mixed models, survival models, and relational event models. The package **bain** (Gu, Hoijtink, Mulder, & Rosseel, 2019) evaluates informed hypotheses for structural equation models. Outside of R, the Fortran 90 program **BIEMS** (Mulder, Hoijtink, & De Leeuw, 2012) evaluates informed hypotheses for multivariate linear models such as MANOVA, repeated measures, and multivariate regression. All these packages rely on one of two implementations of the encompassing prior approach (Klugkist, Kato, & Hoijtink, 2005; Sedransk, Monahan, & Chiu, 1985) to approximate order constrained Bayes factors: the unconditional encompassing method (Klugkist et al., 2005; Hoijtink, 2011; Hoijtink et al., 2008) and the conditional encompassing method (Gu, Mulder, & Deković, 2014; Laudy, 2006; Mulder, 2014; Mulder, 2016; Mulder et al., 2009). Even though the encompassing prior approach is currently the most common method to evaluate informed hypotheses, it becomes increasingly unreliable and inefficient as the number of restrictions increases or the parameter space of the restricted model decreases (Sarafoglou et al., 2021). For instance, simulation studies conducted by Sarafoglou et al., (2021) have illustrated that the unconditional encompassing approach is not able to produce Bayes factors when hypotheses with a large number of constrained parameters are considered (i.e., they considered 18 categories). For hypotheses with fewer categories (i.e., 5 or 6), the method worked well when the data provided either weak or moderate evidence in favor of or against the informed hypothesis. However, when the data provided extreme evidence against the predicted constraints, the method again failed to compute Bayes factors.

As alternative to the encompassing prior approach, Sarafoglou et al., (2021) recently proposed a bridge sampling routine (Bennett, 1976; Meng and Wong, 1996) that computes Bayes factors for informed hypotheses more reliably and efficiently. This routine is implemented in **multibridge** (https://CRAN.R-project.org/package=multibridge) and is suitable to evaluate inequality constraints for multinomial and binomial models as well as combinations between equality and inequality constraints.

Here we showcase how the proposed bridge sampling routine by Sarafoglou et al., (2021) can be performed with **multibridge**. In the remainder of this article, we will introduce the package and its functionalities and describe the methods used to compute the informed hypotheses in binomial and multinomial models. We will illustrate its core functions using three examples and end with a brief discussion and future directions.

## Multibridge

**Fig. 1:**
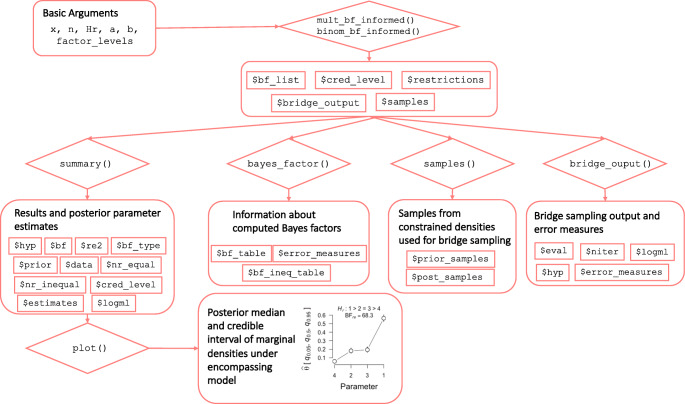
The **multibridge** workflow. The functions mult_bf_ informed or binom_bf_informed return the estimated Bayes factor for the informed hypothesis relative to the encompassing or the null hypothesis. Based on these results different S3 methods can be used to get more detailed information on the individual components of the analysis (e.g., summary, bayes_factor), and parameter estimates of the encompassing distribution (plot)

The general workflow of **multibridge** is illustrated in Fig. 1. The core functions of **multibridge**, that is mult_bf_informed and binom_bf_informed, return the Bayes factor estimate in favor of or against the informed hypothesis. To compute a Bayes factor, the core functions require the observed counts, the informed hypothesis, the parameters of the prior distribution under ${\mathscr{H}}_{e}$, and the category labels. An overview of the basic required arguments of the two core functions are provided in Table 1.

**Table 1:** To estimate the Bayes factor in favor for or against the specified informed hypothesis, the user provides the core functions mult_bf_informed and binom_bf_informed with the basic required arguments listed below

Argument	Description
x	numeric. Vector with data (for multinomial models) or a vector of counts of successes, or a two-dimensional table (or matrix) with 2 columns, giving the counts of successes and failures, respectively (for binomial models).
n	numeric. Vector with counts of trials. Must be the same length as x. Ignored if x is a matrix or a table. Included only in binom_bf_informed.
Hr	string or character. String or a character vector with the user specified informed hypothesis. Parameters may be referenced by the specified factor_levels or by numerical indices.
a	numeric. Vector with concentration parameters of Dirichlet distribution (for multinomial models) or *α* parameters for independent beta distributions (for binomial models). Must be the same length as x. Default sets all parameters to 1.
b	numeric. Vector with *β* parameters. Must be the same length as x. Default sets all *β* parameters to 1. Included only in binom_bf_informed.
factor_levels	character. Vector with category labels. Must be the same length as x.

When calling mult_bf_informed or binom_bf_ informed, the user specifies the data values (x and n for binomial models and x for multinomial models, respectively), the informed hypothesis (Hr), the *α* and *β* parameters of the binomial prior distributions (a and b) or the concentration parameters for the Dirichlet prior distribution (a), respectively, and the category labels of the factor levels (factor_levels). The functions then return the estimated Bayes factor for the informed hypothesis relative to the encompassing hypothesis that imposes no constraints on the category proportions or the null hypothesis which states that all category proportions are equal. Based on these results different S3 methods can be used to get more detailed information on the individual components. For instance, users can extract the Bayes factor with the bayes_factor-method, visualize the posterior parameter estimates under the encompassing hypothesis using the plot-method, or get more detailed information on how the Bayes factor is composed using the summary-method. Table 2 summarizes all S3 methods currently available in **multibridge**.

**Table 2:** S3 methods available in **multibridge**

Function Name(s)	S3 Method	Description
mult_bf_informed, binom_bf_informed	print	Prints model specifications and descriptives.
	summary	Prints and returns the Bayes factor and associated hypotheses for the full model, and all equality and inequality constraints.
	plot	Plots the posterior median and credible interval of the parameter estimates of the encompassing model. Default sets credible interval to 95%.
	bayes_factor	Contains all Bayes factors and log marginal likelihood estimates for inequality constraints.
	samples	Extracts prior and posterior samples from constrained densities (if bridge sampling was applied).
	bridge_output	Extracts bridge sampling output and associated error measures.
	restriction_list	Extracts restriction list and associated informed hypothesis.
mult_bf_inequality, binom_bf_inequality	print	Prints the bridge sampling estimate for the log marginal likelihood and the corresponding percentage error.
	summary	Prints and returns the bridge sampling estimate for the log marginal likelihood and associated error terms.

### Supported hypotheses

**Fig. 2:**
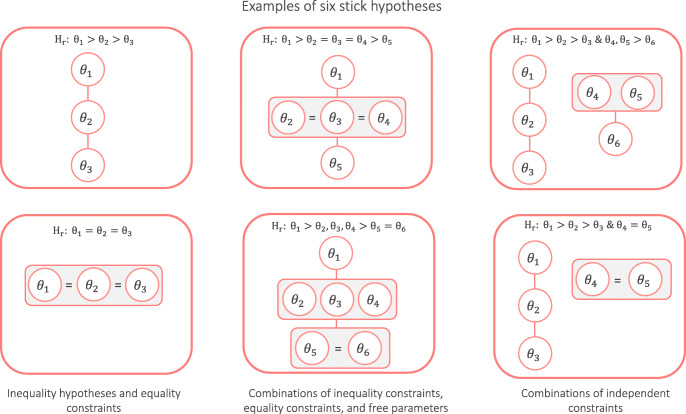
**multibridge** supports informed hypotheses including inequality and equality constraints (left column), combinations of inequality and equality constraints and free parameters (middle column), and multiple independent constraints (right column). Parameters with larger values appear higher in the drawing. A prerequisite of **multibridge** is that all elements within a constraint can be arranged as a linearly ordered set

The following hypotheses are supported in **multibridge**. Users can test hypotheses on equality and inequality constraints among parameters (left column in Fig. 2). We consider inequality constraints, for instance, in Example 3 of this manuscript, when we test whether the probability to violate stochastic dominance decreases for persons with higher education levels (Myung, Karabatsos, & Iverson, 2005).

Additionally, **multibridge** supports the evaluation of combinations of equality constraints, inequality constraints, and free parameters (middle column). As an example, the hypothesis in the top middle panel identifies a largest parameter (*𝜃*_1_) and a smallest parameter (*𝜃*_5_), and equates the remaining parameters (*𝜃*_2_ = *𝜃*_3_ = *𝜃*_4_). Combinations of constraints are considered, for instance, in Example 2 of this manuscript. Based on Nuijten et al., (2016) we test whether the proportion of statistical reporting errors is higher for articles published in the *Journal of Personality and Social Psychology* (JPSP) than for articles published in seven other high-profile psychology journals.

The package also supports the computation of Bayes factors for multiple independent constraints, representing, for instance, two main effects (right column). For instance, the hypothesis in the bottom right panel describes an inequality constraint on the first three category proportions (*𝜃*_1_ > *𝜃*_2_ > *𝜃*_3_) and an equality constraint on the fourth and fifth category proportion (*𝜃*_4_ = *𝜃*_5_).

An important requirement for the hypotheses supported in **multibridge** is that within each independent constraint, all elements are arranged as a linearly ordered set. Elements can refer to individual parameters as shown in the top left panel of Fig. 2. In this example, for each pair of elements one precedes the other in the sequence (i.e., *𝜃*_1_ precedes *𝜃*_2_ and *𝜃*_2_ precedes *𝜃*_3_). Elements can also refer to a group of equality constrained parameters or a group of free parameters as shown in the middle panel of Fig. 2. In the top middle panel, too, for each pair of elements one precedes the other in the sequence (e.g., *𝜃*_1_ precedes (*𝜃*_2_ = *𝜃*_3_ = *𝜃*_4_) and (*𝜃*_2_ = *𝜃*_3_ = *𝜃*_4_) precedes *𝜃*_5_). That is, if the constraint was to be drawn as a Hasse diagram or specified as a character vector, the constrained elements should string together like a chain, ranging from the smallest element to the largest. We refer to these hypotheses as “stick hypotheses”.

**Fig. 3:**
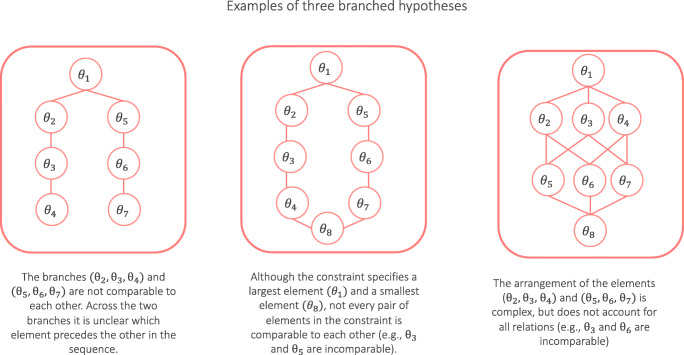
Examples of three hypotheses in which elements in a constraint are arranged as a partial order. In each panel there exist elements that are not comparable with each other, that is, for which neither element precedes the other in the sequence. The partial order shows itself in the branching of the Hasse diagram. These branched hypotheses are currently not supported in **multibridge**

Conversely, “branched hypotheses”, are hypotheses in which elements are not arranged as a linearly ordered set but as a partial order, meaning that some but not all pairs of elements precede one another. These hypotheses are *not* supported in **multibridge**. Examples for branched hypotheses are shown in Fig. 3. For instance, the hypothesis illustrated in the left panel states that *𝜃*_1_ precedes all other parameters. In addition, the hypothesis orders the branches (*𝜃*_2_, *𝜃*_3_, *𝜃*_4_) and (*𝜃*_5_, *𝜃*_6_, *𝜃*_7_). However, it remains unclear whether, for instance, *𝜃*_3_ or *𝜃*_5_ precede the other in the sequence. Thus, not all pairs of elements are comparable. Similarly, in all three examples of branched hypotheses it is unclear whether *𝜃*_3_ precedes or follows *𝜃*_6_. Researchers whose theories give rise to branched hypotheses and wish to test them can do so using one of the alternative R packages, for instance, **multinomineq** by Heck and Davis-Stober (2019).

When an informed hypothesis includes combinations of equality and inequality constraints, the core functions in **multibridge** split the hypothesis to compute Bayes factors separately for imposed equality constraints (for which the Bayes factor has an analytic solution) and inequality constraints (for which the Bayes factor is estimated using bridge sampling). Hence, for hypotheses that include combinations of equality and inequality constraints the bayes_factor method separately returns the Bayes factor for the equality constraints and the conditional Bayes factor for the inequality constraints given the equality constraints.

The informed hypothesis Hr can be conveniently specified as a string or a character vector describing the relations among the category proportions. A simple ordering of three category proportions, *𝜃*_1_ > *𝜃*_2_ > *𝜃*_3_, can be specified either as c("t1", ">", "t2", ">", "t3"), or as "t1 > t2 > t3". To assign labels to the parameters, they must be passed to the argument factor_levels. **multibridge** then assumes that the order within the category labels correspond to the order of the data vector. Alternatively, the informed hypotheses can be specified using indices (e.g., "1 > 2 > 3"). To avoid circularity, an index or category label can be used only once within an informed hypothesis.

Signs permitted to specify informed hypotheses are the "<"-sign and ">"-sign for inequality constraints, the "="-sign for equality constraints, the ","-sign for parameters that vary freely within a constraint, and the “&”-sign to connect multiple independent constraints. For instance, the informed hypothesis in the top right panel in Fig. 2, that is, "t1 > t2 > t3 & t4 , t5 > t6", states that t1 is bigger than t2, and that t2, is bigger than t3. In addition, the hypothesis states that t4 and t5 are bigger than t6, with no further constraints imposed among t4 and t5. x<− c(2 , 1 , 5 , 4 , 1 , 8) n<− c(10 , 7 , 13 , 7 , 9 , 14) a<− b<− c(1 , 1 , 1 , 1 , 1 , 1) #Testing ordinal r e l a t i o n s and #equality constraints mult_bf_informed (x =x ,a =a ,Hr =’1 > 2> 3 ’) mult_bf_informed (x =x ,a =a ,Hr =’1 = 2 = 3 ’) binom_bf_informed (x =x ,n =n ,a =a ,b =b ,Hr =’1 > 2> 3 ’) binom_bf_informed (x =x ,n =n ,a =a ,b =b ,Hr =’1 = 2 = 3 ’) #Testing combinations of ordinal #constraints ,equality constraints ,# and free parameters mult_bf_informed (x =x ,a =a ,Hr =’1 = 2 = 3 > 4> 5 = 6 ’) binom_bf_informed (x =x ,n =n ,a =a ,b =b ,Hr =’1 < 2, 3, 4 < 5 = 6 ’) #Testing combinations of independent #constraints mult_bf_informed (x =x ,a =a ,Hr =’1 > 2> 3& 4, 5 = 6 ’) binom_bf_informed (x =x ,n =n ,a =a ,b =b ,Hr =’1 > 2> 3& 5 = 6 ’)

When testing equality constrained hypotheses, users should be aware that there is a difference between assuming equality of category proportions and adding categories together, that is, the hypothesis ${\mathscr{H}}_{r}: \theta _{1} = \theta _{2} > \theta _{3} = \theta _{4}$ differs from the hypothesis ${\mathscr{H}}_{r}: \theta _{1} + \theta _{2} > \theta _{3} + \theta _{4}$. The first hypothesis concerns four category proportions of which two pairs are expected to be equal; as a result, we assign a *K* = 4 Dirichlet prior to this distribution. The second hypothesis concerns only two categories since we assume that *𝜃*_1_ and *𝜃*_2_ belong to one group and *𝜃*_3_ and *𝜃*_4_ belong to the other. Consequently, one assigns a *K* = 2 Dirichlet prior to this distribution. Therefore, to test the second hypothesis, the respective counts of the categories should first be combined and the analysis should be performed on the basis of these new data. #Merging categories or setting them #equal do not yield the same r e s u l t s #Hr :t1 =t2 < t3 =t4 x<− c(20 , 7 , 5 , 9) a<− c(1 , 1 , 1 , 1) summary (mult_bf_informed (x =x ,a =a ,Hr =’1 = 2 > 3 = 4 ’))∖(bf #Hr :t1 +t2 < t3 +t4 x<− c(20 + 7 , 5 + 9) a<− c(1 , 1) summary (mult_bf_informed (x =x ,a =a ,Hr =’1 > 2 ’))∖) bf

In **multibridge**, the functions mult_bf_informed and binom_bf_informed perform all necessary analysis steps. Other available functions compute Bayes factors for hypotheses that postulate only equality or only inequality constraints, and draw from constrained multinomial distributions and distributions of multiple independent binomials. A list of all currently available functions and data sets is given in Table 3.

**Table 3:** Core functions available in **multibridge**

Function Name(s)	Description
mult_bf_informed	Evaluates informed hypotheses on multinomial parameters.
mult_bf_inequality	Estimates the marginal likelihood of a constrained prior or posterior Dirichlet distribution.
mult_bf_equality	Computes Bayes factor for equality constrained multinomial parameters using the standard Bayesian multinomial test.
mult_tsampling	Samples from constrained prior or posterior Dirichlet density.
lifestresses,peas	Data sets associated with informed hypotheses in multinomial models.
binom_bf_informed	Evaluates informed hypotheses on binomial parameters.
binom_bf_inequality	Estimates the marginal likelihood of constrained prior or posterior beta distributions.
binom_bf_equality	Computes Bayes factor for equality constrained binomial parameters.
binom_tsampling	Samples from constrained prior or posterior beta densities.
journals	Data set associated with informed hypotheses in binomial models.
generate_restriction_list	Encodes the informed hypothesis.

## Methodological background

In this section we provide background information on the methods implemented in **multibridge**. Specifically, we formalize multinomial models and models that feature independent binomial probabilities, and define Bayes factors for the Bayesian multinomial test and testing equality of multiple independent binomial probabilities. Furthermore, the section discusses the influence of priors on the Bayes factors, illustrates how to compute posterior model probabilites and how to compare two informed hypotheses with each other, and provides a non-technical introduction into the bridge sampling routine implemented in **multibridge**. Mathematical details of the methods and principles discussed here can be found in Sarafoglou et al., (2021) and Gronau et al., (2017).

In the binomial model, we assume that the elements in the vector of successes **x** and the elements in the vector of total number of observations **n** in the *K* categories follow independent binomial distributions $\mathbf {x} \sim {\prod }_{k = 1}^{K} \text {Binomial}(\theta _{k}, n_{k})$, where *𝜃*_*k*_ is the *k* th category proportion. From this distribution we can derive the likelihood of the data given the parameters:

$$ p(\mathbf{x} \mid \boldsymbol{\theta}) = \prod\limits_{k=1}^{K} {{n_{k}}\choose{x_{k}}}\theta_{k}^{x_{k}}(1-\theta_{k})^{n_{k}-x_{k}}. $$

The parameter vector of the binomial success probabilities ***𝜃*** contains the underlying category proportions and assume that categories are independent. Therefore, a suitable choice for a prior distribution for ***𝜃*** is a vector of independent beta distributions with parameters ***α*** and ***β***, thus $\boldsymbol {\theta } \sim {\prod }_{k = 1}^{K} \text {Beta}(\alpha _{k}, \beta _{k})$. The prior density is given by:

$$ p(\boldsymbol{\theta}) = \prod\limits_{k=1}^{K} \frac{ \theta_{k}^{\alpha_{k} - 1}(1-\theta_{k})^{\beta_{k} - 1}}{\text{B}(\alpha_{k}\text{, }\beta_{k})}, $$

where B(*α*_*k*_, *β*_*k*_) is the beta function:

$$ \text{B}(\alpha_{k}\text{, }\beta_{k}) = \frac{\Gamma(\alpha_{k}){\Gamma}(\beta_{k})}{\Gamma(\alpha_{k} + \beta_{k})}. $$

The multinomial model generalizes the binomial model for cases where *K* > 2. In this model, we assume that the vector of observations **x** in the *K* categories follows a multinomial distribution in which the parameters of interest, ***𝜃***, represent the underlying category proportions, thus $\mathbf {x} \sim \text {Multinomial}(x_{+}, \boldsymbol {\theta })$, where $x_{+} = {\sum }_{k=1}^{K} x_{k}$.

Since the *K* categories are dependent, the vector of probability parameters is constrained to sum to one, such that ${\sum }_{k = 1}^{K} (\theta _{1}, \cdots , \theta _{K}) = 1$. Therefore, a suitable choice for a prior distribution for ***𝜃*** is the Dirichlet distribution with concentration parameter vector ***α***, $\boldsymbol {\theta } \sim \text {Dirichlet}(\boldsymbol {\alpha }):$

$$ p(\boldsymbol{\theta}) = \frac{1}{\text{B}(\boldsymbol{\alpha})} \prod\limits_{k=1}^{K} \theta_{k}^{\alpha_{k}-1}, $$

where B(***α***) is the multivariate beta function:

$$ \text{B}(\boldsymbol{\alpha}) = \frac{{\prod}_{k = 1}^{K} {\Gamma}(\alpha_{k})}{\Gamma \left( {\sum}_{k = 1}^{K} \alpha_{k} \right)}. $$

In **multibridge**, we have deliberately chosen to leave the priors at the original scale (i.e., in the probability space), because it makes it easier to express ones expectations about data patterns. Alternative approaches transform the model parameters into the probit space, which has the advantage that correlations can be specified for hierarchical models (e.g., as in the latent-trait model for multinomial processing tree models, Klauer^2010^; Matzke, Dolan, Batchelder, and Wagenmakers 2015). However, these transformations make the development of priors more difficult and can lead to unintended consequences; for instance, a uniform prior on the probit scale does not translate to a uniform prior on the probability scale (as discussed in Heck and Wagenmakers^2016^).

### Developing suitable prior distributions

In the binomial and multinomial model, the concentration parameters have an intuitive interpretation. In the binomial model, the parameters *α*_*k*_ can be interpreted as vector of *a priori* successes that observations fall within the various categories and *β*_*k*_ can be interpreted as vector of *a priori* failures. Likewise, in the multinomial model, *α*_*k*_ can be interpreted as vector of *a priori* category counts. It follows, that the higher the number of concentration parameters is, the information the prior contains and the more influence it has on parameter estimation and hypothesis testing.

Developing suitable prior distributions for Bayesian inference is a much discussed topic involving various theoretical and computational considerations (see Consonni, Fouskakis, Liseo, and Ntzoufras 2018 for a review paper on prior distributions for objective Bayesian analysis). Therefore, recommending approaches for developing appropriate prior distributions is, in our view, a difficult undertaking. In this section, we therefore present a selected subset of approaches that we consider particularly suitable for assigning adequate priors for the multiple binomials model and the multinomial model.

**Fig. 4:**
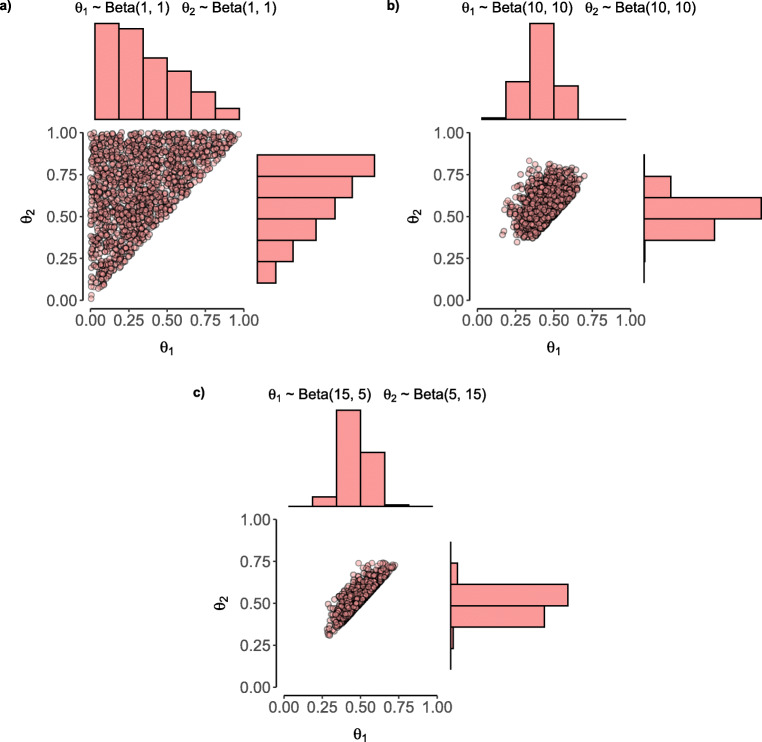
The development of a prior distribution should be accompanied by a visual inspection of the prior predictive. Here we display three prior distributions on two binomial probabilities that are constrained to be *𝜃*_1_ < *𝜃*_2_. The uniform distribution (panel a) assigns equal mass to all permissible values of the constrained space. A symmetric prior (panel b) concentrates the mass in the center of the distribution. A prior describing a constraint in the opposite direction (panel c), puts most of the density along the diagonal

If researchers possess no knowledge or expectations about the plausible parameter values, a uniform distribution can be assigned across the parameter space. This prior assumes that before seeing the data, each category contains one observation, that is, all concentration parameters are set to one. A uniform prior distribution, puts equal probability mass on all permitted parameter values, similar to the adjusted priors for reparametrized models proposed by Heck and Wagenmakers (2016) (see Fig. 4). However, **multibridge** allows priors to be set on the original scale.

We recommend incorporating prior knowledge into the models whenever possible. Based on theories, expert knowledge, or informed guesses, researchers often have expectations about plausible and implausible parameter values. In these cases, the prior should match these expectations (Lee & Vanpaemel, 2018). For instance, in the case of informed hypotheses, prior counts can be chosen to match a particular expected ordinal trend. To determine whether the chosen priors are consistent with the theory, researchers can visualize and assess prior predictive distributions, that is, the distribution of the model parameters and data patterns predicted by the priors (Gabry, Simpson, Vehtari, Betancourt, & Gelman, 2019; Schad, Betancourt, & Vasishth, 2021; Wagenmakers et al., 2021). The developed priors should reflect expectations about the parameters and make sensible predictions. This is particularly important for Bayes factor hypothesis testing; when the purpose is parameter estimation, however, it may be more informative to assign prior parameter distributions that are relatively wide (e.g., Doorn et al.,^2021^).

Furthermore, one can choose the observed category counts of previous studies as priors for the current one, as is often suggested for replication studies and referred to as “Bayesian learning” (e.g., Verhagen & Wagenmakers 2014). This approach constructs highly informative priors; instead of describing the new data as precisely as possible, the goal with this approach is quantify the additional knowledge gained by the new data. Finally, priors can be constructed using a fraction of the likelihood of the data while centering it on the the mean of the parameter range (Gu, Mulder, & Hoijtink, 2018; Mulder, 2014).

### Bayes factor

**multibridge** features two different methods to compute Bayes factors: one method computes Bayes factors for equality constrained parameters (which can be computed analytically) and one method computes Bayes factors for inequality constrained parameters (which needs to be approximated). In cases where informed hypotheses feature combinations between inequality and equality constraints, **multibridge** computes the overall Bayes factor BF_*re*_ by multiplying the individual Bayes factors for both constraint types. This is motivated by the fact that the Bayes factor for combinations (BF_*re*_) will factor into a Bayes factor for the equality constraints (BF_1*e*_) and a conditional Bayes factor for the inequality constraints given the equality constraints (BF_2*e*∣1*e*_). For instance, to evaluate the hypothesis ${\mathscr{H}}_{r}: \theta _{1} > \theta _{2} = \theta _{3}$, **multibridge** factors the Bayes factor as follows:

$$ \text{BF}_{re} = \underbrace{\frac{p(\theta_{1} > \theta_{23} \mid \theta_{2} = \theta_{3} \text{, }\mathbf{x}\text{, } \mathcal{H}_{e})}{p(\theta_{1} > \theta_{23} \mid \theta_{2} = \theta_{3}\text{, } \mathcal{H}_{e})}}_{\text{BF}_{2e \mid 1e}} \times \underbrace{\frac{p(\theta_{2} = \theta_{3} \mid \mathbf{x}\text{, } \mathcal{H}_{e})}{p(\theta_{2} = \theta_{3} \mid \mathcal{H}_{e})}}_{\text{BF}_{1e}}, $$

where the subscript 1 denotes the hypothesis that only features equality constraints, the subscript 2 denotes the hypothesis that only features inequality constraints, and *p*(*𝜃*_1_ > *𝜃*_23_∣*𝜃*_2_ = *𝜃*_3_) refers to a Dirichlet integral, where the category proportions *𝜃*_2_ and *𝜃*_3_ are collapsed. See Sarafoglou et al., (2021) for the proof and a detailed account of this method.

#### Testing equality constraints

For equality constrained binomial models **multibridge** supports two null hypotheses, one stating that all parameters are equal and one stating that all parameters are equal to a specific value. Both null hypotheses are tested against an encompassing hypothesis. Under the encompassing hypothesis, we specify a Beta(*α*_*k*_, *β*_*k*_) prior on each of the *𝜃*_*k*_ that yields the following marginal likelihood:

$$ p(\mathbf{x} \mid \mathcal{H}_{e}) = \frac{{\prod}_{k=1}^{K} {{n_{k}}\choose{x_{k}}} \times \text{B}(x_{k} + \alpha_{k}\text{, }n_{k} - x_{k} + \beta_{k})}{{\prod}_{k=1}^{K} \text{B}(\alpha_{k}\text{, }\beta_{k})}. $$

Under the first null hypothesis which states that all binomial probabilities are set equal without a constraint on a specific value, we collapse all individual Beta(*α*_*k*_, *β*_*k*_) priors and correct for the change in categories; if *K* categories are collapsed, *K* − 1 is subtracted from the concentration parameters. The resulting prior is a Beta(*α*_+_ − (*K* − 1), *β*_+_ − (*K* − 1)) distribution on *𝜃*, where $\alpha _{+} = {\sum }_{k=1}^{K} \alpha _{k}$ and $\beta _{+} = {\sum }_{k=1}^{K} \beta _{k}$. Hence, a Beta(1, 1) prior on each individual category proportion yields again a Beta(1, 1) prior on the categories that are collapsed. When the prior is more informative, say a Beta(2, 2) prior on three individual category proportions, it would result in a Beta(4, 4) prior on *𝜃* as the information available is added together. The corresponding marginal likelihood takes the following form:

$$ p(\mathbf{x} \mid \mathcal{H}_{01}) = \frac{ {\prod}_{k=1}^{K}{{n_{k}}\choose{x_{k}}} \times \text{B}(x_{+} + \alpha_{+} - (K - 1)\text{, }n_{+}-x_{+} +\beta_{+} - (K - 1))}{\text{B}(\alpha_{+} - (K - 1)\text{, }\beta_{+} - (K - 1))}. $$

We can now compute the Bayes factor BF_01*e*_ as follows:

$$ \begin{array}{@{}rcl@{}} \text{BF}_{0e} &=& \frac{p(\mathbf{x} \mid \mathcal{H}_{0})}{p(\mathbf{x} \mid \mathcal{H}_{e})} \\ &=& \frac{\frac{ {\prod}_{k=1}^{K}{{n_{k}}\choose{x_{k}}} \times \text{B}(x_{+} + \alpha_{+} -(K - 1)\text{, }n_{+}-x_{+} +\beta_{+} - (K - 1))}{\text{B}(\alpha_{+} - (K - 1)\text{, }\beta_{+} - (K - 1))} }{\frac{{\prod}_{k=1}^{K} {{n_{k}}\choose{x_{k}}} \times \text{B}(x_{k} + \alpha_{k}\text{, }n_{k} - x_{k} + \beta_{k})}{{\prod}_{k=1}^{K} \text{B}(\alpha_{k}\text{, }\beta_{k})}} \\ &=& \frac{ {\prod}_{k=1}^{K} \text{B}(x_{+} + \alpha_{+} - (K - 1)\text{, }n_{+}-x_{+} +\beta_{+} -(K - 1)) }{{\prod}_{k=1}^{K} \text{B}(x_{k} + \alpha_{k}\text{, }n_{k} - x_{k} + \beta_{k})} \\ &&\times \frac{{\prod}_{k=1}^{K} \text{B}(\alpha_{k}\text{, }\beta_{k})}{\text{B}(\alpha_{+} - (K - 1)\text{, }\beta_{+} - (K - 1))} \end{array} $$

The second null hypothesis states that all binomial probabilities in a model are assumed to be exactly equal *and* equal to a predicted value *𝜃*_0_. Under this hypothesis, the prior reduces to a single point and the marginal likelihood simplifies to the likelihood function:

$$ p(\mathbf{x} \mid \mathcal{H}_{02}) = \theta_{0}^{x_{+}}(1 - \theta_{0})^{n_{+} - x_{+}} \times \prod\limits_{k=1}^{K}{{n_{k}}\choose{x_{k}}}. $$

The Bayes factor for the second null hypothesis is then defined as:

$$ \text{BF}_{02e} = \frac{{\prod}_{k=1}^{K} \text{B}(\alpha_{k} \text{, } \beta_{k})}{{\prod}_{k=1}^{K} \text{B}(\alpha_{k} + x_{k}\text{, } \beta_{k} + n_{k} - x_{k})} \times \theta_{0}^{x_{+}} (1 - \theta_{0})^{n_{+} - x_{+}}. $$

Note that **multibridge** only supports the specification of one predicted value for all binomial probabilities. x <− c(3 , 4 , 10 , 11) n<− c(15 , 12 , 12 , 12) a<− c(1 , 1 , 1 , 1) b<− c(1 , 1 , 1 , 1) #assuming a l l binomial proportions are equal binom_bf_equality (x =x ,n =n ,a =a ,b =b )# assuming a l l binomial proportions are equal #and equal to a predicted value binom_bf_equality (x =x ,n =n ,a =a ,b =b ,p = 0.5)

The Bayes factor BF_0*e*_ for the multinomial test is defined as:

$$ \text{BF}_{0e} = \frac{\text{B}(\boldsymbol{\alpha})}{\text{B}(\boldsymbol{\alpha}+\mathbf{x})}  \times \prod\limits_{k=1}^{K} \theta_{0k}^{x_{k}}, $$

where *𝜃*_0*k*_ represent the predicted category proportions (see Sarafoglou et al., 2021 for the derivation). For multinomial models, under the null hypothesis, category probabilities can either all be set equal (i.e., all category probabilities are $\frac {1}{K}$) or can replaced with the user-specified predicted values. x <− c(3 , 4 , 10 , 11) a<− c(1 , 1 , 1 , 1) #assuming a l l category proportions are #exactly equal mult_bf_equality (x =x ,a =a )# specifying predicted values mult_bf_equality (x =x ,a =a ,p =c (0.1 , 0.1 , 0.3 , 0.5))

#### Testing inequality constraints

For inequality constrained binomial and multinomial models, users can specify informed hypotheses that are either tested against a null hypothesis postulating that all parameters are equal or against the encompassing hypothesis which lets all parameters free to vary. Generally, to obtain the marginal likelihood of the informed hypothesis, it is necessary to integrate over the restricted parameter space, which is difficult to compute. As a solution to the problem of computing marginal likelihood of the informed hypothesis, Klugkist et al., (2005) derived an identity that defines the Bayes factor BF_*re*_ as the ratio of proportions of posterior and prior parameter space consistent with the restriction. This identity forms the basis of the encompassing prior approach. Recently, Sarafoglou et al., (2021) highlighted that these proportions can be reinterpreted as the marginal likelihoods (i.e., the normalizing constants) of the constrained posterior and constrained prior distribution. The prior distribution consistent with the restriction takes the following form:

$$ p(\boldsymbol{\theta} \mid \mathcal{H}_{r}) = \frac{p(\boldsymbol{\theta} \mid \mathcal{H}_{e}) \mathbb{I}(\boldsymbol{\theta}\in\mathcal{R}_{r})}{{\int}_{\mathcal{R}_{e}} p(\boldsymbol{\theta}\mid\mathcal{H}_{r}) \text{d}\boldsymbol{\theta}}, $$

where $\mathbb {I}(\boldsymbol {\theta }\in \mathcal {R}_{r})$ is an indicator function that is one for parameter values in the that obey the constrained and zero otherwise. The constrained posterior distribution of the parameters under the informed hypothesis can be represented in the same way,

$$ p(\boldsymbol{\theta} \mid \mathbf{x}\text{, }\mathcal{H}_{r}) = \frac{p(\boldsymbol{\theta} \mid \mathbf{x}\text{, } \mathcal{H}_{e}) \mathbb{I}(\boldsymbol{\theta}\in\mathcal{R}_{r})}{{\int}_{\mathcal{R}_{e}} p(\mathbf{x} \mid \boldsymbol{\theta}) p(\boldsymbol{\theta}\mid\mathcal{H}_{r}) \text{d}\boldsymbol{\theta}}. $$

The Klugkist identity (Klugkist et al., 2005) can be derived from the marginal likelihoods of the two distributions as follows:

1 $$ \begin{array}{@{}rcl@{}} \text{BF}_{re} &=& \frac{\overbrace{p(\boldsymbol{\theta} \in \mathcal{R}_{r} \mid \mathbf{x}\text{, }\mathcal{H}_{e})}^{\begin{array}{ccc}{\text{\small  Marginal likelihood of}}\\{\small\text{constrained posterior distribution}}\end{array}}}{\underbrace{p(\boldsymbol{\theta} \in \mathcal{R}_{r} \mid \mathcal{H}_{e})}_{\underset{\text{\small\ constrained prior distribution}}{\text{\small\ Marginal likelihood of}}}}. \end{array} $$

The Klugkist identity made it possible to utilize numerical sampling methods such as bridge sampling to compute the Bayes factor. The following section provides a conceptual introduction to bridge sampling and how it is used in the context of evaluating informed hypotheses.

### Bridge sampling routine

The bridge sampling routine implemented in **multibridge** is a numerical method to estimate the marginal likelihood of a target density cf., Gronau et al., (2017) and Overstall and Forster (2010). The identity used in bridge sampling is displayed in Eq. 2; it considers the unnormalized target density, a proposal density with known normalizing constant, and an arbitrary bridge function. The numerator in Eq. 2 describes the expected value of the unnormalized target density evaluated with samples from the proposal density. The denominator is the expected value of the proposal density and a bridge function evaluated with samples from the target density. The bridge function serves the purpose of increasing the overlap between the two densities, thus increasing the efficiency and accuracy of the method. The bridge sampling identity can then be expressed as follows:

2 $$ \begin{array}{@{}rcl@{}} p(\boldsymbol{\theta} \in \mathcal{R}_{r} \mid \mathcal{H}_{e}) = \frac{\mathbb{E}_{g(\boldsymbol{\theta})}\left( p(\boldsymbol{\theta}\mid \mathcal{H}_{e}) \mathbb{I}(\boldsymbol{\theta}\in\mathcal{R}_{r})h(\boldsymbol{\theta})\right)}{\mathbb{E}_{\text{prior}} \left( g(\boldsymbol{\theta})h(\boldsymbol{\theta})\right)}, \end{array} $$

where the term *h*(***𝜃***) refers to the bridge function proposed by Meng and Wong (1996), *g*(***𝜃***) refers to a proposal density (in this application we choose the multivariate normal density), and $p(\boldsymbol {\theta }\mid {\mathscr{H}}_{e}) \mathbb {I}(\boldsymbol {\theta }\in \mathcal {R}_{r})$ is the unnormalized target density; in this case it represents the part of the prior parameter space under the encompassing hypothesis that is in accordance with the constraint. In the conventional application of bridge sampling, the marginal likelihoods of the two competing hypotheses are estimated, that is, the marginal likelihood of the informed hypothesis and the marginal likelihood of the encompassing hypothesis. But on the basis of Eq. 1, the routine implemented in **multibridge** estimates the marginal likelihood of the restricted prior and restricted posterior density.

It should be noted that the bridge sampling algorithm implemented in **multibridge** is an adapted version of the algorithm implemented in the R package **bridgesampling** (Gronau, Singmann, & Wagenmakers, 2020) and allows for the specification of informed hypotheses on probability vectors.^1^

**Fig. 5:**
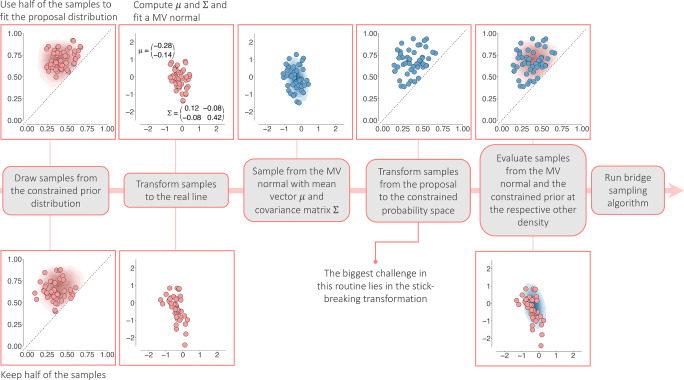
A schematic illustration of the steps taken to estimate the marginal likelihood of the constrained prior distribution of two binomial probabilities under ${\mathscr{H}}_{r}: \theta _{1} < \theta _{2}$. As starting point, the routine requires samples from the constrained prior distribution (red). Following a transformation to the real line, a multivariate normal distribution (blue) is fit to half of the samples. The results from evaluating the samples from the multivariate normal distribution and the constrained prior distribution at the respective other density are needed to compute the expected values displayed in Eq. 2. As final step, the bridge sampling algorithm estimates the marginal likelihood of the constrained prior distribution using an iterative scheme

A schematic representation of the bridge sampling routine is displayed in Fig. 5. To estimate the marginal likelihood, bridge sampling requires samples from the target distribution, that is, the constrained Dirichlet distribution for multinomial models and constrained beta distributions for binomial models, and samples from the proposal distribution which in principle can be any distribution with a known marginal likelihood; in **multibridge** the proposal distribution is the multivariate normal distribution. Samples from the target distribution are generated using the Gibbs sampling algorithms proposed by Damien and Walker (2001). For binomial models, we apply the suggested Gibbs sampling algorithm for constrained beta distributions. In the case of the multinomial models, we apply an algorithm that simulates values from constrained Gamma distributions which are then transformed into Dirichlet random variables. To sample efficiently from these distributions, **multibridge** provides a C++ implementation of this algorithm. Samples from the proposal distribution are generated using the standard rmvnorm-function from the R package **mvtnorm** (Genz et al., 2020).

Despite the bridge function, the efficiency of the bridge sampling method is optimal only if the target and proposal distribution operate on the same parameter space and have sufficient overlap. We therefore probit transform the samples of the constrained distributions to move the samples from the probability space to the entire real line. Subsequently, we use half of these draws to construct the proposal distribution using the method of moments. Then, samples are drawn from the proposal density and transformed back into the probability space, ensuring that the samples correspond to the informed hypothesis. These transformed samples are then used to evaluate the unnormalized target density.

The numerator in Eq. 2 evaluates the unnormalized density for the constrained prior distribution with samples from the proposal distribution. The denominator evaluates the normalized proposal distribution with samples from the constrained prior distribution. Since the optimal bridge function proposed by Meng and Wong (1996) contains the marginal likelihood of the target density –the quantity we wish to compute– an iterative scheme is applied to obtain the estimate. **multibridge** then runs the iterative scheme until the tolerance criterion suggested by Gronau et al., (2017) is reached. The sampling from the target and proposal distribution, the transformations and computational steps are performed automatically within the core functions of **multibridge**. The user only needs to provide the functions with the data, a prior and a specification of the informed hypothesis. As part of the standard output of binom_bf_informed and mult_bf_informed, the functions return the bridge sampling estimate for the log marginal likelihood of the target distribution, its associate relative mean square error and the number of iterations needed to until the bridge sampling estimator reached the tolerance criterion.

To summarize, in order to implement the bridge sampling method we only need to be able to sample from the constrained densities. Crucially, when using bridge sampling, it does not matter how small the constrained parameter space is in proportion to the encompassing density. This gives the method a decisive advantage over the encompassing prior approach in terms of accuracy and efficiency especially (1) when binomial and multinomial models with moderate to high number of categories (i.e., *K* > 10) are evaluated and (2) when relatively little posterior mass falls in the constrained parameter space.

### Stick-breaking transformation

**Fig. 6:**
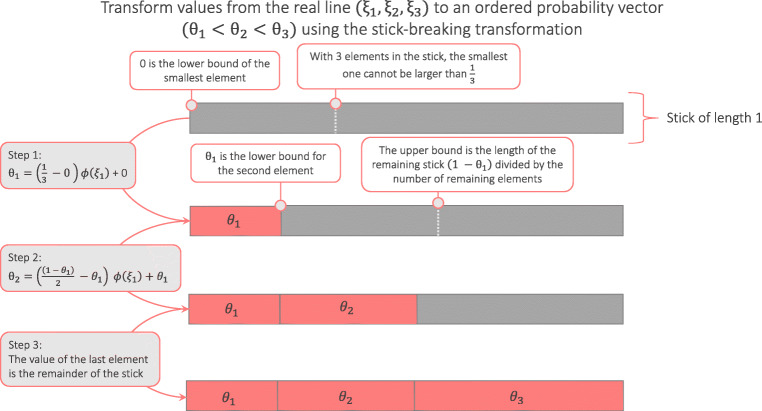
The stick-breaking transformation of elements on the real line to the ordered probability space. The stick-breaking transformation moves from the smallest to the largest element to determine its lower and upper bounds

The bridge sampling routine in **multibridge** uses the multivariate normal distribution as proposal distribution, which requires moving samples from target distribution to the real line and conversely, moving samples from the real line to the ordered probability space. Crucially, the transformation needs to retain the ordering of the parameters, that is, it needs to take into account the lower bound and the upper bound of each parameter. Elements from the real line to the ordered probability space are then transformed as follows:

$$ \theta_{k} = (u_{k} -l_{k}) {\Phi}(\xi_{k})+l_{k}, $$

where *ξ*_*k*_ is *k* th the element on the real line, Φ is the cumulative density function of a standard normal and *u*_*k*_ and *l*_*k*_ are the upper and lower bounds of *ξ*_*k*_, respectively. The largest element is simply the remainder of the stick. The inverse transformation is given by

$$ \xi_{k} = {\Phi}^{-1}\left( \frac{\theta_{k} - l_{k}}{u_{k} - l_{k}}\right), $$

where Φ^− 1^ denote the inverse cumulative density function. To determine the bounds, **multibridge** uses a probit transformation, as proposed in Wagenmakers et al., (2021), which transforms the elements by moving from the smallest to the largest value. A schematic illustration of the stick-breaking transformation is given in Fig. 6, detailed technical details of the transformation are provided in the Appendix.

To perform the transformation from a parameter vector on the real line to an ordered probability vector, we need to determine the lower and upper bound of each parameter. Consider an increasing trend of four parameters, that is, *𝜃*_1_ < *𝜃*_2_ < *𝜃*_3_. The lower bound for for the smallest element in the parameter vector, *𝜃*_1_, is 0. For *𝜃*_2_ and *𝜃*_3_ the lower bound is the preceding element in the vector. That is, the lower bound for *𝜃*_2_ is *𝜃*_1_, lower bound for *𝜃*_3_ is *𝜃*_2_.

This definition holds for both binomial models and multinomial models. Differences in these two models appear only when determining the upper bound for each parameter. For binomial models, the upper bound for each parameter is 1. For multinomial models, due to the sum-to-one constraint the upper bounds need to be computed differently. As proposed in Frigyik, Kapila, and Gupta (2010) and Stan Development Team (2020) we represent ***𝜃*** as unit-length stick which we subsequently divide into as many elements as there are parameters in the constraint (Stan Development Team, 2020). In this approach, the upper bounds are derived from on the values of smaller elements as well as on the number of remaining larger parameters in the stick. Concretely, for the smallest element in the parameter vector, *𝜃*_1_, the upper bound is $\frac {1}{3}$; if this element were larger than that it would be impossible to create a probability vector with increasing values. For *𝜃*_2_ and *𝜃*_3_ the upper bound is the proportion of the unit-length stick that has not yet been accounted for in the transformation divided by the number of parameters in the remaining stick. For instance, the upper bound for *𝜃*_2_ is defined as $\frac {1 - \theta _{1}}{2}$. This transformation allows us to effectively transform elements from the real line to an constrained probability space and is therefore a main component of the bridge sampling algorithm.

The drawback of this transformation is, however, that it can only be performed if all elements in the constraint are arranged as a linearly ordered set, thus, only works for “stick hypotheses”. For hypotheses in which elements in a constraint are arranfed as a partial order, the assumption is violated that for a given parameter smaller elements and the number of parameters in the remaining stick determine their upper bound.

### Poster model probabilites, and Bayes factor transitivity

Consider a scenario where researchers entertain more than two hypotheses that they wish to compare. For instance, they may entertain two informed hypotheses ${\mathscr{H}}_{r1}$ and ${\mathscr{H}}_{r2}$ as well as a null hypothesis ${\mathscr{H}}_{0}$ and the encompassing hypothesis ${\mathscr{H}}_{e}$. An overview of the relative plausibility of all *M* = 4 models simultaneously may be obtained by presenting the posterior model probabilities for all hypotheses, $p({\mathscr{H}}_{i} | x)$, *i* = 1,⋯ ,4 (Berger and Molina, 2005). Posterior model probabilities are not automatically computed in **multibridge**; however, after computing the individual Bayes factors, the posterior model probabilities can be obtained easily. Denoting the prior model probability for hypothesis ${\mathscr{H}}_{r1}$ by $p({\mathscr{H}}_{r1})$, the posterior model probability $p({\mathscr{H}}_{r1} \mid \mathbf {x})$ is given by:

$$ p(\mathcal{H}_{r1} \mid \mathbf{x}) = \frac{\frac{p(\mathbf{x} \mid \mathcal{H}_{r1})}{p(\mathbf{x} \mid \mathcal{H}_{e})} \times p(\mathcal{H}_{r1})}{\displaystyle\sum\limits_{i = 1}^{M} \frac{p(\mathbf{x} \mid \mathcal{H}_{i})}{p(\mathbf{x} \mid \mathcal{H}_{e})} \times p(\mathcal{H}_{i})}. $$

When all hypotheses are equally likely *a priori*, this simplifies to:

$$ p(\mathcal{H}_{r1} \mid \mathbf{x}) = \frac{\text{BF}_{r1e}}{\text{BF}_{r1e} + \text{BF}_{r2e} + \text{BF}_{0e} + \text{BF}_{ee}}, $$

where BF_*ee*_ equals 1. In R, the posterior model probabilities can be computed as follows: #posterior model probability of Hr1 #given three alternative hypotheses p_Hr1_x <− bfr1e /(bfr1e +bfr2e +bf0e + 1) #bfee = 1

Posterior model probabilities are useful for comparing multiple hypotheses; however, they are relative quantities that change depending on which other hypotheses are included in the comparison. Thus, hypotheses that describe the data poorly may have high posterior model probabilities if the other hypotheses in the comparison set provide even worse descriptions of the data. In order to gain insight into whether a hypothesis describes the data adequately, we therefore consider so-called bookend hypotheses along with theory-informed hypotheses. That is, we include a hypothesis that maximally constrains the parameter space (such as a point-null hypothesis ${\mathscr{H}}_{0}$) and the encompassing hypothesis ${\mathscr{H}}_{e}$ that does not constrain the parameter space (in this case, that makes no ordinal predictions, Lee & Vanpaemel 2018). A hypothesis is then considered adequate if it outperforms these two bookend models.

In addition to posterior model probabilities, Bayes factors can also be calculated directly between two informed hypotheses. The comparison of any two informed hypotheses with one another follows from the fact that Bayes factors are transitive. For instance, the Bayes factor comparison between two informed hypotheses ${\mathscr{H}}_{r1}$ and ${\mathscr{H}}_{r2}$ can be obtained by first computing BF_*r*1*e*_ and BF_*r*2*e*_, and then dividing out the common hypothesis ${\mathscr{H}}_{e}$:

$$ \text{BF}_{r1r2} = \frac{\text{BF}_{r1e}}{\text{BF}_{r2e}}. $$

For this comparison to be feasible, the encompassing hypotheses must be identical, that is, the same prior distribution must be assigned to the category proportions.

### Prior sensitivity

Bayesian hypothesis testing has been criticised as the priors exert too much influence on the Bayes factors (e.g., Kass & Raftery 1995). That is, even if the data are informative enough to overwhelm the prior for parameter estimation, priors can still influence the Bayes factors. The development of suitable priors is thus an important part of Bayesian hypothesis testing.

But even priors that are justified by theory are to a certain degree arbitrary. For instance, if one expects an increasing trend in the data, the parameters in the prior can be chosen to reflect that trend. The exact number of *a priori* category counts, however, is at the discretion of the analyst. It is therefore considered good research practice to conduct a sensitivity analysis on the final results (e.g., Lee & Vanpaemel, 2018; Sinharay & Stern, 2002; Vanpaemel, 2010). In a sensitivity analysis, a set of plausible priors are determined in addition to the prior chosen in the main analysis for which the Bayes factors are calculated. The range of Bayes factors then gives an indication of the extent to which the results are fragile or robust to different modeling choices. In general, the prior on which the final analysis is performed as well as the set of priors used to conduct the sensitivity analysis should be determined and preregistered before seeing the data to ensure a fair comparison of the hypotheses of interest.

## Usage and examples

In the following, we will outline three examples on how to use **multibridge** to compare an informed hypothesis to a null or encompassing hypothesis. The first example concerns multinomial data and the second and third example concerns independent binomial data. Additional examples are available as vignettes (see vignette(package = “multibridge”)).

The two core functions of **multibridge**—mult_bf_ informed and the binom_bf_informed—can be illustrated schematically as follows: mult_bf_informed (x ,Hr ,a ,f a c t o r _ l e v e l s )binom_bf_informed (x ,n ,Hr ,a ,b ,f a c t o r _ l e v e l s )

### Example 1: applying a Benford test to greek fiscal data

The first-digit phenomenon, otherwise known as Benford’s law (Benford, 1938; Newcomb, 1881) states that the expected proportion of leading digits in empirical data can be formalized as follows: for any given leading digit *d*,*d* = (1,⋯ ,9) the expected proportion is approximately equal to

$$ \mathbb{E}_{\theta_{d}}= \log_{10}((d + 1)/d). $$

This means that in an empirical data set, numbers with smaller leading digits are more common than numbers with larger leading digits. Specifically, a number has leading digit 1 in 30.1*%* of the cases, and leading digit 2 in 17.61*%* of the cases,leading digit 9 is the least frequent digit with an expected proportion of only 4.58*%* (see Table 4 for an overview of the expected proportions). Empirical data for which this relationship holds include population sizes, death rates, baseball statistics, atomic weights of elements, and physical constants (Benford, 1938). In contrast, artificially generated data, such as telephone numbers, do in general not obey Benford’s law (Hill, 1995). Given that Benford’s law applies to empirical data but not artificially generated data, a so-called Benford test can be used in fields like accounting and auditing to check for indications for poor data quality (for an overview, see e.g., Durtschi, Hillison, and Pacini (2004), Nigrini (2012), Nigrini and Mittermaier (1997)). Data that do not pass the Benford test, should raise audit risk concerns, meaning that it is recommended that they undergo additional follow-up checks (Nigrini, 2019).

**Table 4:** Observed counts, observed proportions, and expected proportions of first digits in the Greek fiscal data set. The total sample size was *N* = 1,497 observations. Note that the observed proportions and counts deviate slightly from those reported in Rauch et al., (2011) (probably due to rounding errors)

Leading digit	Observed Counts	Observed Proportions	Expected Proportions: Benford’s Law
1	509	0.340	0.301
2	353	0.236	0.176
3	177	0.118	0.125
4	114	0.076	0.097
5	77	0.051	0.079
6	77	0.051	0.067
7	53	0.035	0.058
8	73	0.049	0.051
9	64	0.043	0.046

Below we discuss four possible Bayesian adaptations of the Benford test. In a first scenario we simply conduct a Bayesian multinomial test in which we test the point-null hypothesis ${\mathscr{H}}_{0}$ which predicts a Benford distribution. In a second scenario we test the informed hypothesis ${\mathscr{H}}_{r1}$, which predicts a decreasing trend in the proportions of leading digits. The hypothesis ${\mathscr{H}}_{r1}$ exerts considerably more constraint than ${\mathscr{H}}_{e}$ and provides a more sensitive test if our primary goal is to test whether data comply with Benford’s law or whether the data follow a similar but different trend. In the next two scenarios, our main goal is to identify fabricated data. The third scenario therefore tests the null hypothesis against the hypothesis that all proportions occur equally often. This hypothesis ${\mathscr{H}}_{r2}$ could be considered if it is suspected that the data were generated randomly or could serve as a bookend comparison hypothesis as it maximally constraints the parameter space. In a fourth scenario we test a hypothesis which predicts a trend that is characteristic for manipulated data. This hypothesis, which we denote as ${\mathscr{H}}_{r3}$, could be derived from empirical research on fraud or be based on observed patterns from former fraud cases. For instance, Hill (1995) instructed students to produce a series of random numbers; in the resulting data the proportion of the leading digit 1 occurred most often and the digits 8 and 9 occurred least often which is consistent with the general pattern of Benford’s law. However, the proportion for the remaining leading digits were approximately equal. Note that the predicted distribution derived from Hill (1995) is not currently used as a test to detect fraud, however, for the sake of simplicity, we assume that this pattern could be an indication of manipulated auditing data. All hypotheses will be tested against the encompassing hypothesis ${\mathscr{H}}_{e}$, which too serves as a bookend comparison hypothesis, and which imposes no constraints on the proportion of leading digits.

#### Data and hypothesis

**Fig. 7:**
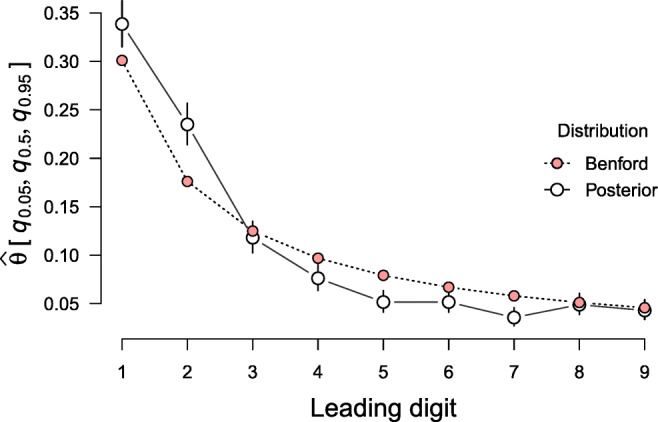
Predictions from Benford’s law (in pink) show together with the posterior medians (black circles) for the category proportions estimated under the encompassing model ${\mathscr{H}}_{e}$. The circle skewers show the 95% credible intervals. Only three of nine intervals encompass the expected proportions, suggesting that the data do not follow Benford’s law. This plot was created using the plot-S3-method for summary.bmult objects in **multibridge**

The data we use to illustrate the computation of Bayes factors were originally published by the European statistics agency “Eurostat” and served as basis for reviewing the adherence to the Stability and Growth Pact of EU member states. Rauch, Göttsche, Brähler, and Engel (2011) conducted a Benford test on data related to budget deficit criteria, that is, public deficit, public debt and gross national products. The data used for this example features the proportion of first digits from Greek fiscal data in the years between 1999 and 2010; a total of *N* = 1,497 numerical data were included in the analysis. We choose this data, since the Greek government deficit and debt statistics states has been repeatedly criticized by the European Commission in this time span (European Commision 2004, 2010). In particular, the commission has accused the Greek statistical authorities to have misreported deficit and debt statistics. For further details on the data set see Rauch et al., (2011). The observed and expected proportions are displayed in Table 4; the expected proportions versus the posterior parameter estimates under the encompassing hypothesis are displayed in Fig. 7.

In this example, the parameter vector of the multinomial model, *𝜃*_1_,⋯ ,*𝜃*_*K*_, reflects the probabilities of a leading digit in the Greek fiscal data being a number from 1 to 9. Each of the hypotheses above will be tested against the encompassing hypothesis ${\mathscr{H}}_{e}$ which imposes no constraints on the parameters. The hypotheses introduced above can then be formalized as follows:

$$ \begin{array}{@{}rcl@{}} \mathcal{H}_{e} &:& \boldsymbol{\theta} \sim \text{Dirichlet}(\mathbf{1}) \\ \mathcal{H}_{0} &\!:\!& \boldsymbol{\theta}_{0} = (0.301, 0.176, 0.125, 0.097, 0.079, 0.067, 0.058, 0.051, 0.046), \\ \mathcal{H}_{r1} &:& \theta_{1} > \theta_{2} > \theta_{3} > \theta_{4} > \theta_{5} > \theta_{6} > \theta_{7} > \theta_{8} > \theta_{9} \\ \mathcal{H}_{r2} &:& \boldsymbol{\theta}_{0} = \left( \frac{1}{9}, \frac{1}{9}, \frac{1}{9}, \frac{1}{9}, \frac{1}{9}, \frac{1}{9}, \frac{1}{9}, \frac{1}{9}, \frac{1}{9}\right)\\ \mathcal{H}_{r3} &:& \theta_{1} > (\theta_{2} = \theta_{3} = \theta_{4} = \theta_{5} = \theta_{6} = \theta_{7}) > (\theta_{8}, \ \theta_{9}). \end{array} $$

#### Method

Both BF_0*e*_ and BF_*r*2*e*_ may be readily computed by means of a Bayesian multinomial test which is implemented in the function mult_bf_equality. This function requires (1) a vector with observed counts, (2) a vector with concentration parameters of the Dirichlet prior distribution under ${\mathscr{H}}_{e}$, and (3) the vector of expected proportions under ${\mathscr{H}}_{0}$ and under ${\mathscr{H}}_{r2}$. In this example, we do not incorporate specific expectations about the distribution of leading digits in the Greek fiscal data and therefore assign a uniform Dirichlet distribution to the proportion of leading digits. That is, we set all concentration parameters under ${\mathscr{H}}_{e}$ to 1 (i.e., we assign ***𝜃*** a uniform Dirichlet prior distribution). This prior supports all possible points equally, meaning that, if the data were completely random, none of the hypotheses under consideration should be favored over the other. #Observed counts x<− c(509 , 353 , 177 , 114 , 77 , 77 ,53 , 73 , 64)# Prior s p e c i f i c a t i o n for Dirichlet# prior distribution under H_ea <− c (1 , 1 , 1 , 1 , 1 , 1 , 1 , 1 , 1)# Expected proportions for H_0 and H_r2p0 <− log10 ((1:9 + 1)/1:9)pr2 <− c (1/9 , 1/9 , 1/9 , 1/9 , 1/9 ,1/9 , 1/9 , 1/9 , 1/9)# Execute the analysisresults_H0_He <− mult_bf_equality (x = x ,a = a , p = p0)results_Hr2_He <− mult_bf_equality (x = x ,a = a , p = pr2 )logBFe0 <− results_H0_He∖ t e x t i t {bf}LogBFe0logBFer2 <− results_Hr2_He∖ t e x t i t {bf}LogBFe0

The hypotheses ${\mathscr{H}}_{r1}$ and ${\mathscr{H}}_{r3}$ contain inequality constraints, and this necessitates the use of the function mult_bf_informed to compute the Bayes factors BF_*r*1*e*_ and BF_*r*3*e*_. This function requires (1) a vector with observed counts, (2) a vector with concentration parameters of the Dirichlet prior distribution under ${\mathscr{H}}_{e}$, (3) labels for the categories of interest (i.e., leading digits), and (4) the informed hypothesis ${\mathscr{H}}_{r1}$ or ${\mathscr{H}}_{r3}$ (e.g., as a string). In addition to the basic required arguments, we use two additional arguments here. The first argument sets the Bayes factor type, that is, whether the output should print the Bayes factor in favor of the informed hypothesis (i.e., BF_*re*_) or in favor of the encompassing hypothesis (i.e., BF_*er*_). It is also possible to compute the log Bayes factor in favor of the hypothesis, which is the setting we choose for this example. The purpose of the second argument seed is to make the results reproducible: #Observed counts x<− c(509 , 353 , 177 , 114 , 77 , 77 ,53 , 73 , 64)# Prior s p e c i f i c a t i o n for Dirichlet# prior distribution under H_ea <− c (1 , 1 , 1 , 1 , 1 , 1 , 1 , 1 , 1)# Labels for categories of i n t e r e s t f a c t o r _ l e v e l s <− 1:9# Specifying the informed hypotheses as# a stringHr1 <− c (’1 > 2 > 3 > 4 > 5 > 6 > 7 > 8 > 9 ’)Hr3 <− c (’1 > 2 = 3 = 4 = 5 = 6 = 7 > 8 , 9 ’)# Execute the analysisresults_He_Hr1 <− mult_bf_informed (x = x ,Hr = Hr1 , a = a , f a c t o r _ l e v e l s = factor_levels ,bf_type = ’LogBFer ’ , seed = 2020)results_He_Hr3 <− mult_bf_informed (x = x ,Hr = Hr3 , a = a , f a c t o r _ l e v e l s = factor_levels ,bf_type = ’LogBFer ’ , seed = 2020)logBFer1 <− summary(results_He_Hr1 )∖(bflogBFer3 <− summary(results_He_Hr3 )∖) bf

We also compute the posterior model probabilities for all hypotheses. The results are shown in Table 5.

**Table 5:** Prior model probabilities, posterior model probabilities, and Bayes factors for five rival accounts of first digit frequencies in the Greek fiscal data set

Hypothesis	$p({\mathscr{H}}_{.})$	$p({\mathscr{H}}_{.} \mid \mathbf {x})$	$\log (\text {BF}_{.e})$
${\mathscr{H}}_{0}$	0.2	1.27 × 10^− 11^	− 17.67
${\mathscr{H}}_{r1}$	0.2	0.9994	7.42
${\mathscr{H}}_{e}$	0.2	0.0006	0
${\mathscr{H}}_{r3}$	0.2	5.97 × 10^− 79^	− 172.70
${\mathscr{H}}_{r2}$	0.2	2.71 × 10^− 212^	− 479.73

The results indicate strong support for ${\mathscr{H}}_{r1}$ –the model in which the proportions are assumed to decrease monotonically– over all other models. The log Bayes factor of ${\mathscr{H}}_{r1}$ against the encompassing hypothesis ${\mathscr{H}}_{e}$ is 7.42, which equates to a Bayes factor of 1,664 on a natural scale.

The strong Bayes factor support for ${\mathscr{H}}_{r1}$ translates to a relatively extreme posterior model probability of 0.9994. By comparison, the posterior model probabilities for hypotheses ${\mathscr{H}}_{r2}$ and ${\mathscr{H}}_{r3}$, that is, the bookend null-hypothesis and the hypothesis predicting a data pattern typical of fraud, are only slightly greater than zero. The posterior model probability for ${\mathscr{H}}_{e}$ is 0.0006. Thus, hypothesis ${\mathscr{H}}_{r1}$ can outperform the two bookend hypotheses ${\mathscr{H}}_{r2}$ and ${\mathscr{H}}_{e}$. That ${\mathscr{H}}_{r1}$ outperforms the unconstrained model ${\mathscr{H}}_{e}$ demonstrates how a parsimonious model that makes precise predictions can be favored over a model that is more complex (e.g., Jefferys and Berger 1992).

#### Sensitivity analysis

In a sensitivity analysis we will determine whether our results are robust against different prior choices. In the main analysis we chose a uniform Dirichlet distribution on the category proportions as prior under ${\mathscr{H}}_{e}$. This prior assigns equal probability to all possible parameter values, but alternative prior distributions are seem also conceivable. Audit researchers may argue for the development of more informative and theory-driven priors that resemble one of the hypotheses under consideration. The Dirichlet parameters vectors specified below resemble the four hypotheses, assuming *N* = 54 prior observations. #Alternative prior s p e c i f i c a t i o n s #Benford ’ s law a0 <− c (16 , 10 , 7 , 5 , 4 , 3 , 3 , 3 , 2)# Monotonically decreasing trenda1 <− c (10 , 9 , 8 , 7 , 6 , 5 , 4 , 3 , 2)# Equal proportionsa2 <− c (6 , 6 , 6 , 6 , 6 , 6 , 6 , 6 , 6)# Fraud patterna3 <− c (12 , 6 , 6 , 6 , 6 , 6 , 6 , 3 , 3)

The sensitivity analysis is then carried out for each prior choice and will be compared to the main results. For this analysis, we are particularly interested in the Bayes factors of the hypothesis postulating a decreasing trend ${\mathscr{H}}_{r1}$ and Benford’s law ${\mathscr{H}}_{0}$ to the encompassing hypothesis ${\mathscr{H}}_{e}$. #Sensitivity analysis for log (BFe_r1 )s e n s i t i v i t y 0 <− mult_bf_informed (x =x ,Hr =Hr1 ,a =a0 ,f a c t o r _ l e v e l s =factor_levels ,bf_type =’ LogBFer ’ , seed = 2020) s e n s i t i v i t y 1 <− mult_bf_informed (x =x ,Hr =Hr1 ,a =a1 ,f a c t o r _ l e v e l s =factor_levels ,bf_type =’ LogBFer ’ , seed = 2020) s e n s i t i v i t y 2 <− mult_bf_informed (x =x ,Hr =Hr1 ,a =a2 ,f a c t o r _ l e v e l s =factor_levels ,bf_type =’ LogBFer ’ , seed = 2020) s e n s i t i v i t y 3 <− mult_bf_informed (x =x ,Hr =Hr1 ,a =a3 ,f a c t o r _ l e v e l s =factor_levels ,bf_type =’ LogBFer ’ , seed = 2020) #Sensitivity analysis for log (BFe_0 )s e n s i t i v i t y 4 <− mult_bf_equality (x =x ,a =a0 ,p =p0 )s e n s i t i v i t y 5 <− mult_bf_equality (x =x ,a =a1 ,p =p0 )s e n s i t i v i t y 6 <− mult_bf_equality (x =x ,a =a2 ,p =p0 )s e n s i t i v i t y 7 <− mult_bf_equality (x =x ,a =a3 ,p =p0 )

The results of the sensitivity analysis are displayed in Table 6. The general direction of the sensitivity analysis agrees with our conclusions drawn from the main analysis. That is, for the Bayes factors of ${\mathscr{H}}_{r1}$ compared to ${\mathscr{H}}_{e}$, the evidence points towards the informed hypothesis. However, the prior exerts an influence on BF_*r*1*e*_; the evidence in favor for the informed hypothesis ranges from weak to extreme evidence. Specifically, when we choose priors that resemble a decreasing trend for the frequency of leading digits, as we did with ***α***_***0***_ and ***α***_***1***_, the Bayes factor becomes smaller and the evidence weak (i.e., (BF_*r*1*e*_∣***α***_***0***_) = 1.87 on the natural scale) and moderate (i.e., (BF_*r*1*e*_∣***α***_***1***_) = 4.74 on the natural scale). When the prior contrasts the data, the evidence becomes very strong or extreme. Thus, a prior that closely resembles the predictive trend reduces to some degree the diagnostic value of the data.

**Table 6:** Results of a sensitivity analysis for the Greek fiscal data set

Description	Prior	$\log (\text {BF}_{r1e})$	$\log (\text {BF}_{0e})$
Uniform	***α***_***e***_ = (1, 1, 1, 1, 1, 1, 1, 1, 1)	7.42	− 17.67
Benford’s law	***α***_***0***_ = (16, 10, 7, 5, 4, 3, 3, 3, 2)	0.63	− 26.00
Montonically decreasing	***α***_***1***_ = (10, 9, 8, 7, 6, 5, 4, 3, 2)	1.56	− 20.94
Centered on mean	***α***_***2***_ = (6, 6, 6, 6, 6, 6, 6, 6, 6)	7.53	− 11.35
Fraud pattern	***α***_***3***_ = (12, 6, 6, 6, 6, 6, 6, 3, 3)	3.93	− 18.62

By contrast, the Bayes factors for ${\mathscr{H}}_{0}$ compared to ${\mathscr{H}}_{e}$ are robust against different prior settings. Here too, the prior changes the Bayes factor estimate but in all cases the data suggests overwhelming evidence in favor of the encompassing hypothesis over Benford’s law.

To summarize, the data offer overwhelming support for hypothesis ${\mathscr{H}}_{r1}$, which postulates a decreasing trend in the digit proportions. This model outperformed both simpler models (e.g., the Benford model and the bookend null-hypothesis) and a more complex model in which the proportions were free to vary. The results are sensitive to our prior choices as a sensitivity analysis showed: for moderately informative priors which resemble the predicted decreasing trend, the ${\mathscr{H}}_{r1}$ cannot outperform the encompassing model. On the other hand, the conclusion that Benford’s law does not offer a good description of the data was robust to different prior settings. Detailed follow-up analyses are needed to discover why the Greek fiscal data fail to adhere to Benford’s law (Nigrini, 2019).

### Example 2: prevalence of statistical reporting errors

This section illustrates how **multibridge** may be used to evaluate models for independent binomial data rather than multinomial data. Our example concerns the prevalence of statistical reporting errors across eight different psychology journals. In any article that uses null hypothesis significance testing, there is a chance that the reported test statistic and degrees of freedom do not match the reported *p*-value, possibly because of copy-paste errors. To flag these errors, Epskamp and Nuijten (2014) developed the R package **statcheck**, which scans the PDF of a given scientific article and automatically detects statistical inconsistencies. This package allowed (Nuijten et al., 2016) to estimate the prevalence of statistical reporting errors in the field of psychology. In total, the authors investigated a sample of 30,717 articles (which translates to over a quarter of a million *p*-values) published in eight major psychology journals between 1985 to 2013: *Developmental Psychology* (DP), the *Frontiers in Psychology* (FP), the *Journal of Applied Psychology* (JAP), the *Journal of Consulting and Clinical Psychology* (JCCP), *Journal of Experimental Psychology: General* (JEPG), the *Journal of Personality and Social Psychology* (JPSP), the *Public Library of Science* (PLoS), *Psychological Science* (PS).

Based on several background assumptions, Nuijten et al., (2016) predicted that the proportion of statistical reporting errors is higher for articles published in the *Journal of Personality and Social Psychology* (JPSP) than for articles published in the seven other journals.

#### Data and hypothesis

Here we reuse the original data published by Nuijten et al., (2016), which we also distribute with the package **multibridge** under the name journals. data (journals )

The (Nuijten et al., 2016) hypothesis of interest, ${\mathscr{H}}_{r}$, states that the prevalence for statistical reporting errors is higher for JPSP than for the other journals.^2^ We will consider two specific versions of the Nuijten et al., (2016) ${\mathscr{H}}_{r}$ hypothesis. The first hypothesis, ${\mathscr{H}}_{r1}$, stipulates that JPSP has the highest prevalence of reporting inconsistencies, whereas the other seven journals share a prevalence that is lower. The second hypothesis, ${\mathscr{H}}_{r2}$, also stipulates that JPSP has the highest prevalence of reporting inconsistencies, but does not commit to any particular structure on the prevalence for the other seven journals.

The **multibridge** package can be used to test ${\mathscr{H}}_{r1}$ and ${\mathscr{H}}_{r2}$ against the null hypothesis ${\mathscr{H}}_{0}$ that all eight journals have the same prevalence of statistical reporting errors. In addition, we will compare ${\mathscr{H}}_{r1}$, ${\mathscr{H}}_{r2}$, and ${\mathscr{H}}_{0}$ against the encompassing hypothesis ${\mathscr{H}}_{e}$ that makes no commitment about the prevalence of reporting inconsistencies across the eight journals. In this example, the parameter vector of the binomial success probabilities, ***𝜃***, reflects the probabilities that articles contain at least one statistical reporting inconsistency across journals. Thus, the above hypotheses can be formalized as follows:

$$ \begin{array}{@{}rcl@{}} \mathcal{H}_{e} &:& \theta_{\text{JAP}} {\cdots} \theta_{\text{JPSP}} \sim \prod\limits_{k = 1}^{K} \text{Beta}(\alpha_{k}, \beta_{k}) \\ \mathcal{H}_{0} &:& \theta_{\text{JAP}} = \theta_{\text{PS}} = \theta_{\text{JCCP}} = \theta_{\text{PLOS}} = \theta_{\text{DP}} = \theta_{\text{FP}}= \theta_{\text{JEPG}} = \theta_{\text{JPSP}}\\ \mathcal{H}_{r1} &:& (\theta_{\text{JAP}} = \theta_{\text{PS}} = \theta_{\text{JCCP}} = \theta_{\text{PLOS}} = \theta_{\text{DP}} = \theta_{\text{FP}}= \theta_{\text{JEPG}}) < \theta_{\text{JPSP}} \\ \mathcal{H}_{r2} &:& (\theta_{\text{JAP}} , \theta_{\text{PS}} , \theta_{\text{JCCP}} , \theta_{\text{PLOS}} , \theta_{\text{DP}} , \theta_{\text{FP}} , \theta_{\text{JEPG}}) < \theta_{\text{JPSP}}. \end{array} $$

#### Method

To compute the Bayes factor BF_0*r*_ we need to specify (1) a vector with observed successes (i.e., the number of articles that contain a statistical inconsistency), (2) a vector containing the total number of observations (i.e., the number of articles), (3) a vector with prior parameter *α*_*k*_ for each binomial proportion of the beta prior distribution under ${\mathscr{H}}_{e}$, (4) a vector with prior parameter *β*_*k*_ for each binomial proportion of the beta prior distribution under ${\mathscr{H}}_{e}$, (5) the category labels (i.e., journal names), and (6) the informed hypothesis ${\mathscr{H}}_{r1}$ or ${\mathscr{H}}_{r2}$ (e.g., as a string). We also change the Bayes factor type to LogBFr0 so that the function returns the log Bayes factor in favor for the informed hypothesis compared to the null hypothesis. Since we have no specific expectations about the distribution of statistical reporting errors in any given journal, we set all parameters *α*_*k*_ and *β*_*k*_ to one which corresponds to uniform beta distributions. With this information, we can now conduct the analysis with the function binom_bf_informed (Table 7). #Since percentages are rounded to two #decimal values ,we round the a r t i c l e s #with an error to obtain integer values x<− round (journals ∖(articles_with_NHST *(journals ∖) perc_articles_with_errors /100))# Total number of a r t i c l e s n <− journals ∖(articles_with_NHST# Prior s p e c i f i c a t i o n for beta# prior distributions under H_ea <− c (1 , 1 , 1 , 1 , 1 , 1 , 1 , 1)b <− c (1 , 1 , 1 , 1 , 1 , 1 , 1 , 1)# Labels for categories of i n t e r e s t journal_names <− journals ∖) journal# Specifying the informed HypothesisHr1 <− c (’JAP = PS = JCCP = PLOS = DP =FP = JEPG < JPSP ’ ) Hr2 <− c (’JAP , PS , JCCP , PLOS , DP ,FP , JEPG < JPSP ’ ) # Execute the analysis for Hr1results_H0_Hr1 <− binom_bf_informed (x =x , n = n , Hr = Hr1 , a = a , b = b , f a c t o r _ l e v e l s = journal_names ,bf_type = ’LogBFr0 ’ , seed = 2020)# Execute the analysis for Hr2results_H0_Hr2 <− binom_bf_informed (x =x , n = n , Hr = Hr2 , a = a , b = b , f a c t o r _ l e v e l s = journal_names ,bf_type = ’LogBFr0 ’ , seed = 2020) LogBFe0 <− results_H0_Hr1 ∖(b f _ l i s t ∖) bf0_ table [ [ ’ LogBFe0 ’ ] ] LogBFr10 <− summary (results_H0_Hr1 )∖(bf LogBFr20 <− summary (results_H0_Hr2 )∖) bf

**Table 7:** Prior model probabilities, posterior model probabilities, and Bayes factors for four hypotheses concerning the prevalence of statistical reporting errors across psychology journals

Hypothesis	$p({\mathscr{H}}_{.})$	$p({\mathscr{H}}_{.} \mid \mathbf {x})$	$\log (\text {BF}_{.0})$
${\mathscr{H}}_{0}$	0.25	1.6073 × 10^− 69^	0
${\mathscr{H}}_{r2}$	0.25	0.8814	158.28
${\mathscr{H}}_{e}$	0.25	0.1186	156.27
${\mathscr{H}}_{r1}$	0.25	1.9517 × 10^− 37^	73.88

As the evidence is extreme in all four cases, we again report all Bayes factors on the log scale. The Bayes factor $\log (\text {BF}_{r20})$ indicates overwhelming evidence for the informed hypothesis that JPSP has the highest prevalence for statistical reporting inconsistencies compared to the null hypothesis that the statistical reporting errors are equal across all eight journals; $\log (\text {BF}_{r20}) =$ 158.28.

For a clearer picture about the ordering of the journals we can investigate the posterior distributions for the prevalence rates obtained under the encompassing model. plot (summary (results_H0_Hr2 ) , xlab =” Journal ”)

**Fig. 8:**
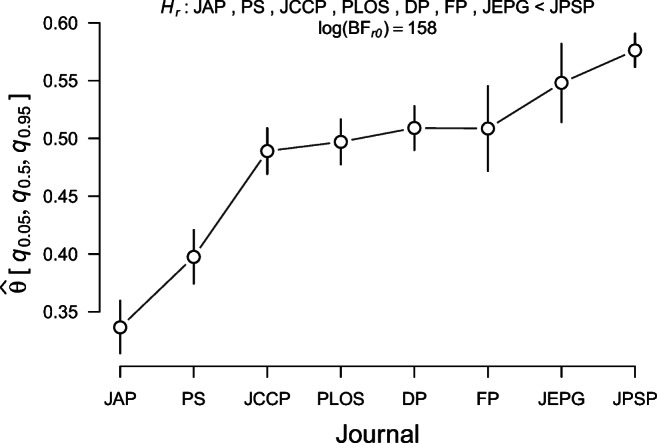
Posterior medians for the prevalence of statistical reporting inconsistencies across eight psychology journals, as obtained using the encompassing model. The circle skewers show the 95% credible intervals. Analysis based on data from Nuijten et al., (2016). This plot was created using the plot-S3-method for summary.bmult objects

The posterior medians and 95% credible intervals are returned by the summary-method and are shown in Fig. 8. The figure strongly suggests that the prevalence of reporting inconsistencies is not equal across all eight journals. This impression may be quantified by comparing the null hypothesis ${\mathscr{H}}_{0}$ to the encompassing hypothesis ${\mathscr{H}}_{e}$. The corresponding Bayes factor equals $\log (\text {BF}_{e0}) =$ 156.27, which confirms that the data dramatically undercut the null hypothesis that the prevalence of statistical reporting inconsistencies is equal across journals.

The data offer most support for the Nuijten hypothesis ${\mathscr{H}}_{r2}$, which posits that JPSP has the highest prevalence but does not commit to any restriction on the prevalences for the remaining seven journals. This hypothesis may be compared to the encompassing hypothesis ${\mathscr{H}}_{e}$, which yields $\log (\text {BF}_{r2e}) =$ 2.01. This means that the observed data are $\exp (2.01) \approx 7.45$ times more likely under ${\mathscr{H}}_{r2}$ than under ${\mathscr{H}}_{e}$; this is moderate evidence for the restriction suggested by Nuijten et al., (2016). Under equal prior probability for the models, this Bayes factor translates to a posterior probability on ${\mathscr{H}}_{e}$ of 0.119, an amount that researchers may deem too large to discard in an all-or-none fashion.

To summarize, the data provide moderate evidence for the hypothesis stated by Nuijten et al., (2016) thatthe prevalence of statistical reporting inconsistencies in JPSP is higher than that in seven other psychology journals.

### Example 3: effects of gender and education on the violation of stochastic dominance

This section illustrates the comparison of four nested hypotheses concerning independent binomial probabilities. In his study, Birnbaum (1999) presented new possibilities of online testing for psychological science (in the late 1990s online testing was still novel and rarely used). To compare data collected from an online research to traditional lab research, Birnbaum (1999) collected experimental data from 1224 participants online and 124 participants in the lab. In his experiment participants played 20 rounds of a gambling game. In each round, they were presented with two gambles with different probabilities and monetary values and were asked to indicate which gamble they would rather play. The gamble chosen by the participants was then played once. Birnbaum (1999) examined the characteristics of the two samples, for instance, in terms of their risk aversion and their consistency with decision making axioms, such as stochastic violations, and correlated them with different demographics.

The author analyzed the proportion of stochastic violations for different demographic variables, noting a seemingly ordinal pattern for the probabilities to violate of stochastic dominance for the factors gender (m=male, f=female) and education (1=doctorate degree, 2=postgraduate degree, 3=bachelor’s degree, 4=less than bachelor’s degree). In a later study, Myung et al., (2005) presented a Bayesian inference framework to test decision making axioms (using the “Bayesian *p*-value”) and used Birnbaum’s data as an example on how to assess violations of stochastic dominance and their relationship with covariates. Concretely, Myung et al., (2005) reanalyzed the data from Birnbaum (1999) and tested the informed hypothesis that stochastic dominance is violated more frequently in women compared to men and more frequently in lower education levels than higher education levels.

#### Data and hypothesis

We will use data from Birnbaum (1999) as presented in Myung et al., (2005). The data show the stochastic violations of the online sample for one of the gambling rounds featuring 1212 valid responses (see Table 8). dat <− data .frame (gender =rep (c (’ male ’ , ’ female ’ ) , each = 4) , education =rep (c (’1 ’ , ’2 ’ , ’3 ’ , ’4 ’) , 2) , l e v e l s =paste0 (rep (c (’ m’ , ’ f’ ) , each = 4) , 1:4) , violation =c (0.487 , 0.477 , 0.523 , 0.601 , 0.407 , 0.555 , 0.650 , 0.622) , n= c(80 , 88 , 195 , 163 , 54 , 108 , 206 , 318) , x= c(39 , 42 , 102 , 98 , 22 , 60 , 134 , 198))

**Table 8:** Observed counts and observed proportions of stochastic dominance violations for the N = 1,212 participants in Birnbaum (1999)

Education	Observed Counts	Observed Proportions
Male		
Doctorate Degree	39/80	0.49
Postgraduate Degree	42/88	0.48
Bachelor’s Degree	102/195	0.52
Less than Bachelor’s degree	98/163	0.60
Female		
Doctorate Degree	22/54	0.41
Postgraduate Degree	60/108	0.56
Bachelor’s Degree	134/206	0.65
Less than Bachelor’s degree	198/318	0.62

The data are split by gender and education level of the participants

The parameter vector of the binomial success probabilities, *𝜃*_1_,⋯ ,*𝜃*_*K*_, contains the probabilities of observing a value in a particular category; here, it reflects the probabilities of violating stochastic dominance for a particular subgroup (e.g., women with a doctorate). We will compare three inequality-constrained hypotheses ${\mathscr{H}}_{r1}$, ${\mathscr{H}}_{r2}$, ${\mathscr{H}}_{r3}$ formulated by Myung et al., (2005). The first hypothesis ${\mathscr{H}}_{r1}$ encodes the main effect for gender and states that the probability to violate stochastic dominance is lower for men than for women. The second hypothesis ${\mathscr{H}}_{r2}$ encodes the main effect of education and states that the probability to violate stochastic dominance is lower for persons with higher education levels. The third hypothesis ${\mathscr{H}}_{r3}$ combines hypotheses ${\mathscr{H}}_{r1}$ and ${\mathscr{H}}_{r2}$. We will test this hypothesis against the encompassing hypothesis ${\mathscr{H}}_{e}$ without any constraints. In addition, we will include a bookend null-hypothesis ${\mathscr{H}}_{0}$ predicting that all probabilities are equal. The set of candidate hypotheses can therefore be written as follows:

$$ \begin{array}{@{}rcl@{}} \mathcal{H}_{e} &:& (\theta_{\text{m1}}, \theta_{\text{m2}}, \theta_{\text{m3}}, \theta_{\text{m4}}, \theta_{\text{f1}}, \theta_{\text{f2}}, \theta_{\text{f3}}, \theta_{\text{f4}}) \\ \mathcal{H}_{0} &:& \boldsymbol{\theta}_{0} = \left( \frac{1}{8}, \frac{1}{8}, \frac{1}{8}, \frac{1}{8}, \frac{1}{8}, \frac{1}{8}, \frac{1}{8}, \frac{1}{8}\right), \\ \mathcal{H}_{r1} &:& (\theta_{\text{m1}}, \theta_{\text{m2}}, \theta_{\text{m3}}, \theta_{\text{m4}}) < (\theta_{\text{f1}}, \theta_{\text{f2}}, \theta_{\text{f3}}, \theta_{\text{f4}})\\ \mathcal{H}_{r2} &:& (\theta_{\text{m1}}, \theta_{\text{f1}}) < (\theta_{\text{m2}}, \theta_{\text{f2}}) < (\theta_{\text{m3}}, \theta_{\text{f3}}) < (\theta_{\text{m4}}, \theta_{\text{f4}}) \\ \mathcal{H}_{r3} &:& \theta_{\text{m1}} < \theta_{\text{f1}} < \theta_{\text{m2}} < \theta_{\text{f2}} < \theta_{\text{m3}} < \theta_{\text{f3}} < \theta_{\text{m4}} < \theta_{\text{f4}}. \end{array} $$

#### Method

To evaluate the inequality-constrained hypothesis, we need to specify (1) a vector with observed successes, and (2) a vector containing the total number of observations, (3) the informed hypothesis, (4) a vector with prior parameters alpha for each binomial proportion, (5) a vector with prior parameters beta for each binomial proportion, and (6) the labels of the categories of interest (i.e., gender and education level). As with the previous two example, we assign a uniform Beta prior to the binomial probabilities. #number of violations x<− dat ∖ $x #total number people in the category n<− dat ∖ $n #Specifying the informed hypotheses #null hypothesis p0 <− c(1/8 , 1/8 , 1/8 , 1/8 , 1/8 , 1/8 , 1/8 , 1/8) #informed hypotheses Hr1 <− c(’ m1 ,m2 ,m3 ,m4 < f1 ,f2 ,f3 ,f4 ’ ) Hr2 <− c(’ m1 ,f1 < m2 ,f2 < m3 ,f3 < m4 ,f4 ’ ) Hr3 <− c(’ m1 < f1 < m2 < f2 < m3 < f3 < m4 < f4 ’ ) #Prior s p e c i f i c a t i o n #We assign a uniform beta #distribution to each binomial propotion a<− c(1 , 1 , 1 , 1 , 1 , 1 , 1 , 1) b<− c(1 , 1 , 1 , 1 , 1 , 1 , 1 , 1) #categories of i n t e r e s t gender_edu <− dat ∖ $ l e v e l s

With this information, we can now conduct the analysis with the function binom_bf_informed(). Since we are interested in quantifying evidence in favor of the informed hypotheses compared to the encompassing hypothesis, we set the Bayes factor type to BFre. For reproducibility, we are also setting a seed. results_H0_He <− mult_bf_equality (x =x ,a =a ,p =p0 )results_Hr1_He <− binom_bf_informed (x =x ,n =n ,Hr =Hr1 ,a =a ,b =b ,f a c t o r _ l e v e l s =gender_edu ,bf_type =’ BFre ’ , seed = 2020) results_Hr2_He <− binom_bf_informed (x =x ,n =n ,Hr =Hr2 ,a =a ,b =b ,f a c t o r _ l e v e l s =gender_edu ,bf_type =’ BFre ’ , seed = 2020) results_Hr3_He <− binom_bf_informed (x =x ,n =n ,Hr =Hr3 ,a =a ,b =b ,f a c t o r _ l e v e l s =gender_edu ,bf_type =’ BFre ’ , seed = 2020)

The results are summarized in Table 9. We first inspect the Bayes factors for the three informed hypotheses compared to the encompassing hypothesis. For hypotheses ${\mathscr{H}}_{r1}$, the data suggest moderate evidence for the encompassing hypothesis compared to the informed hypothesis, with a Bayes factor of 6.43. This hypothesis predicted a main effect of gender, that is, men should have a lower probability of violating stochastic dominance than women regardless of their education level. For hypotheses ${\mathscr{H}}_{r2}$ and ${\mathscr{H}}_{r3}$, the data provide strong evidence in favor of the informed hypothesis compared to the encompassing hypothesis, with Bayes factors of 17.82 and 22.36, respectively. However, there is no predictive advantage of ${\mathscr{H}}_{r3}$ over ${\mathscr{H}}_{r2}$; the Bayes factor directly comparing these hypotheses is $\text {BF}_{r3r2} = \frac {\text {BF}_{r3e}}{\text {BF}_{r2e}} =$ 1.26. The degree to which the data conforms to the predicted pattern from ${\mathscr{H}}_{r3}$ becomes apparent when we plot the posterior estimates. plot (summary (results_Hr3_He ))

**Table 9:** Prior model probabilities, posterior model probabilities, and Bayes factors for four hypotheses concerning the relationship between gender and education level on the probability of violating stochastic dominance

Hypothesis	$p({\mathscr{H}}_{.})$	$p({\mathscr{H}}_{.} \mid \mathbf {x})$	BF_.*e*_
${\mathscr{H}}_{e}$	0.25	0.0242	1
${\mathscr{H}}_{0}$	0.25	1.34 × 10^− 53^	5.55 × 10^− 52^
${\mathscr{H}}_{r1}$	0.25	0.0038	0.16
${\mathscr{H}}_{r2}$	0.25	0.4310	17.82
${\mathscr{H}}_{r3}$	0.25	0.5410	22.36

To compare all four hypotheses directly with each other, we computed the posterior model probabilities: post_probs <− data .frame (Hyps =c (’ p(He |x ) ’ , ’ p(H0 |x ) ’ , ’ p(Hr1 |x ) ’ , ’ p(Hr2 |x ) ’ , ’ p(Hr3 |x ) ’) , Prob =c (1 , BF0e ,BFr1e ,BFr2e ,BFr3e )/ sum (c (1 , BF0e ,BFr1e ,BFr2e ,BFr3e )))

The model that predicts only a gender effect performs worse than the baseline model without any restrictions. Hypothesis ${\mathscr{H}}_{r3}$ outperforms all other models, including the bookend hypotheses, with a posterior model probability of 54%. Here too, however, the posterior probability of hypothesis ${\mathscr{H}}_{r2}$ is with 43% almost as high as ${\mathscr{H}}_{r3}$. To sum up, even though ${\mathscr{H}}_{r3}$ yield the biggest Bayes factor and the highest posterior model probability, the difference advantage to ${\mathscr{H}}_{r2}$ is slim. Note that (Myung et al., 2005) and (Birnbaum, 1999) concluded that hypothesis ${\mathscr{H}}_{r3}$ performs the best. In contrast, our analysis suggested that here is strong evidence for an effect of education, but it is inconclusive whether the effect is moderated by gender.

## Discussion

The R package **multibridge** facilitates the estimation of Bayes factors for informed hypotheses in both multinomial and independent binomial models. The efficiency gains of **multibridge** are particularly pronounced when the parameter restrictions are highly informative or when the number of categories is large.

**multibridge** supports the evaluation of informed hypotheses that feature equality constraints, inequality constraints, and free parameters, as well as combinations between them. Moreover, users can choose to test the informative hypothesis against an encompassing hypothesis that lets all parameters vary freely or against the null hypothesis that states that category proportions are exactly equal. Beyond the core functions currently implemented in **multibridge**, there are several natural extensions we aim to include in future versions of this package. For instance, to compare several models with each other we plan to implement functions that compute the posterior model probabilities. Another extension is to facilitate the specification of hierarchical binomial and multinomial models which would allow users to analyze data where responses are nested within a higher-order structure such as participants, schools, or countries. Hierarchical multinomial models can be found, for instance, in source memory research where people need to select a previously studied item from a list (e.g., Arnold, Heck, Bröder, Meiser, and Boywitt 2019); a hierarchical binomial model was applied, for instance, in Hoogeveen, Sarafoglou, and Wagenmakers (2020), to evaluate laypeople’s accuracy in predicting replication outcomes for social science studies.

Furthermore, to make the method accessible to a larger audience of users and students, the informed Bayesian multinomial test and the informed Bayesian test for multiple binomials will be made available in future versions of the software package JASP (JASP Team, 2022). JASP offers an intuitive graphical user interface and does not require extensive knowledge in programming.

In addition, we plan to expand the types of hypotheses that can be evaluated in future versions of this package. Currently, **multibridge** only supports informed hypotheses which are “stick hypotheses”, that is, hypotheses in which all elements within a constraint are linearly ordered. While the quantity shown in Eq. 1 admits in principle any constraint imposed on a vector of category proportions, this requirement is necessary for the bridge sampling routine, in order to transform samples from the real line to the probability space. To be able to evaluate more general ordinal constrains including “branch-hypotheses” with bridge sampling in the future, the stick-breaking transformation needs to be further refined. Arguably, this refinement can be realized more easily for transformations of multiple binomials than for multinomials, since independent binomials live in probability space but are not constrained by the sum-to-one condition.

Finally, we aim to enable the specification of more general informed hypotheses, including hypotheses on the size ratios of the parameters (e.g., *𝜃*_1_ < 2 × *𝜃*_2_) or on their odds ratios $\left (\text {e.g.,} \frac {\theta _{1}}{(\theta _{1} + \theta _{2})} < \frac {\theta _{3}}{(\theta _{3} + \theta _{4})}\right )$. A framework to evaluate these constraints using the unconditional encompassing approach has already been proposed (Klugkist, Laudy, & Hoijtink, 2010). We believe that the bridge sampling method could also be extended to test these hypotheses as in principle, as all the building blocks are already in place. Specifically, **multibridge** takes size ratios into account when it evaluates hypotheses featuring combinations of equality and inequality constraints. For these hypotheses, **multibridge** first evaluates the equality constraints separately and then evaluates the inequality constraints given the equality constraints hold. To do so, the algorithm merges equality-constrained categories but tracks their initial number to effectively sample from the constrained parameter space and to transform the parameters. For odds ratios, on the other hand, a suitable sampling method and transformation has not yet been developed. To facilitate the evaluation of these hypotheses, alternative methods to sample and transform the parameters are required.

## Data Availability

The source code of the R package is available at: https://github.com/ASarafoglou/multibridge/. In addition, readers can access the code for reproducing all analyses and plots via our project folder on the Open Science Framework: https://osf.io/2wf5y/.

## Appendix

### Transforming an ordered probability vector to the real line

The bridge sampling routine in **multibridge** uses the multivariate normal distribution as proposal distribution, which requires moving the target distribution ***𝜃*** to the real line. Crucially, the transformation needs to retain the ordering of the parameters, that is, it needs to take into account the lower bound *l*_*k*_ and the upper bound *u*_*k*_ of each *𝜃*_*k*_. To meet these requirements, **multibridge** uses a probit transformation, as proposed in Sarafoglou et al., (2021), and subsequently transforms the elements in ***𝜃***, moving from its lowest to its highest value. In the binomial model, we move all elements in ***𝜃*** to the real line and thus construct a new vector $\boldsymbol {y} \in \mathbb {R}^{K}$. For multinomial models it follows from the sum-to-one constraint that the vector ***𝜃*** is completely determined by its first *K* − 1 elements, where *𝜃*_*K*_ is defined as $1 - {\sum }_{k = 1}^{K-1} \theta _{k}$. Hence, for multinomial models we will only consider the first *K* − 1 elements of ***𝜃*** and we will transform them to *K* − 1 elements of a new vector $\boldsymbol {y} \in \mathbb {R}^{K - 1}$.

Let *ϕ* denote the density of a normal variable with a mean of zero and a variance of one, Φ denote its cumulative density function, and Φ^− 1^ denote the inverse cumulative density function. Then for each element *𝜃*_*k*_, the transformation is

$$ \xi_{k} = {\Phi}^{-1}\left( \frac{\theta_{k} - l_{k}}{u_{k} - l_{k}}\right), $$

The inverse transformation is given by

$$ \theta_{k} = (u_{k} - l_{k}) {\Phi}(\xi_{k}) + l_{k}. $$

To perform the transformations, we need to determine the lower bound *l*_*k*_ and the upper bound *u*_*k*_ of each *𝜃*_*k*_. Assuming *𝜃*_*k*− 1_ < *𝜃*_*k*_ for *k* ∈{2⋯ ,*K*} the lower bound for any element in ***𝜃*** is defined as

$$ \begin{array}{@{}rcl@{}} l_{k} = \left\{\begin{array}{ll} 0 & \text{if } k = 1 \\ \theta_{k - 1} & \text{if } 1 < k < K. \end{array}\right. \end{array} $$

This definition holds for both binomial models and multinomial models. Differences in these two models appear only when determining the upper bound for each parameter. For binomial models, the upper bound for each *𝜃*_*k*_ is simply 1. For multinomial models, however, due to the sum-to-one constraint the upper bounds depend on the values of smaller elements as well as on the number of remaining larger elements in ***𝜃***. To be able to determine the upper bounds, we represent ***𝜃*** as unit-length stick which we subsequently divide into *K* elements (Stan Development Team, 2020). By using this so-called stick-breaking method we can define the upper bound for any *𝜃*_*k*_ as follows:

C1 $$ \begin{array}{@{}rcl@{}} u_{k} = \left\{\begin{array}{ll} \frac{1}{K} & \text{if } k = 1 \\ \frac{1 - {\sum}_{i < k} \theta_{i}}{ERS} & \text{if } 1 < k < K, \end{array}\right. \end{array} $$

where $1 - {\sum }_{i < k} \theta _{i}$ represents the length of the remaining stick, that is, the proportion of the unit-length stick that has not yet been accounted for in the transformation. The elements in the remaining stick are denoted as *ERS*, and are computed as follows:

$$ ERS = K - 1 + k. $$

The transformations outlined above are suitable only for ordered probability vectors, that is, for informed hypotheses in binomial and multinomial models that only feature inequality constraints. However, when informed hypotheses also feature equality constrained parameters, as well as parameters that are free to vary we need to modify the formula. Specifically, to determine the lower bounds for any *𝜃*_*k*_, we need to take into account how many parameters were set equal to it (denoted as *e*_*k*_) and how many parameters were set equal to its preceding value *𝜃*_*k*− 1_ (denoted as *e*_*k*− 1_):

C2 $$ \begin{array}{@{}rcl@{}} l_{k} = \left\{\begin{array}{ll} 0 & \text{if } k = 1 \\ \frac{\theta_{k - 1}}{e_{k-1}} \times e_{k} & \text{if } 1 < k < K. \end{array}\right. \end{array} $$

The upper bound for parameters in the binomial models still remains 1. To determine the upper bound for multinomial models we must, additionally for each element *𝜃*_*k*_, take into account the number of free parameters that share common upper and lower bounds (denoted with *f*_*k*_). The upper bound is then defined as:

C3 $$ \begin{array}{@{}rcl@{}} u_{k} = \left\{\begin{array}{ll} \frac{1 - (f_{k} \times l_{k})}{K} = \frac{1}{K} & \text{if } k = 1 \\ \left( \frac{1 - {\sum}_{i < k} \theta_{i} - (f_{k} \times l_{k})}{ERS} \right) \times e_{k} & \text{if } 1 < k < K \text{ and } u_{k} \geq \max(\theta_{i < k}), \\ \left( 2 \times \left( \frac{1 - {\sum}_{i < k} \theta_{i} - (f_{k} \times l_{k})}{ERS} \right)\right. \\\left.- \max(\theta_{i < k}) \right) \times e_{k} & \text{if } 1 < k < K \text{ and } u_{k} < \max(\theta_{i < k}). \end{array}\right. \end{array} $$

The elements in the remaining stick are then computed as follows

$$ ERS = e_{k} + \sum\limits_{j > k} e_{j} \times f_{j}. $$

The rationale behind these modifications will be described in more detail in the following sections. In **multibridge**, information that is relevant for thetransformation of the parameter vectors is stored in the generated restriction_list which is returned by the main functions binom_bf_informed and mult_bf_informed but can also be generated separately with the function generate_restriction_list. This restriction list features the sublist inequality_constraints which encodes the number of equality constraints collapsed in each parameter in nr_mult_equal. Similarlythe number of free parameters that share common bounds are encoded under nr_mult_free.

#### Equality constrained parameters

In cases where informed hypotheses feature a mix of equality and inequality constrained parameters, we compute the Bayes factor BF_*r**e*_, by multiplying the individual Bayes factors for both constraint types with each other:

$$ \text{BF}_{re} = \text{BF}_{1e} \times \text{BF}_{2e} \mid \text{BF}_{1e}, $$

where the subscript 1 denotes the hypothesis that only features equality constraints and the subscript 2 denotes the hypothesis that only features inequality constraints. To receive BF_2*e*_∣BF_1*e*_, we collapse all equality constrained parameters in the constrained prior and posterior distributions into one category. This collapse has implications on the performed transformations.

When transforming the samples from the collapsed distributions, we need to account for the fact that the inequality constraints imposed under the original parameter values might not hold for the collapsed parameters. Consider, for instance, a multinomial model in which we specify the following informed hypothesis

$$ \mathcal{H}_{r}: \theta_{1} < \theta_{2} = \theta_{3} = \theta_{4} < \theta_{5} < \theta_{6}, $$

where samples from the encompassing distribution take the values (0.05, 0.15, 0.15, 0.15, 0.23, 0.27). For these parameter values the inequality constraints hold since 0.05 is smaller than 0.15, 0.23, and 0.27. However, the same constraint does not hold when we collapse the categories *𝜃*_2_, *𝜃*_3_, and *𝜃*_4_ into *𝜃*_∗_. That is, the collapsed parameter *𝜃*_∗_ = 0.15 + 0.15 + 0.15 = 0.45 is now larger than 0.23 and 0.27. In general, to determine the lower bound for a given parameter *𝜃*_*k*_ we thus need to take into account both the number of collapsed categories in the preceding parameter *e*_*k*− 1_ as well as the number of collapsed categories in the current parameter *e*_*k*_. Thus, lower bounds for the parameters need to be adjusted as follows:

$$ \begin{array}{@{}rcl@{}} l_{k} = \left\{\begin{array}{ll} 0 & \text{if } k = 1 \\ \frac{\theta_{k - 1}}{e_{k-1}} \times e_{k} & \text{if } 1 < k < K, \end{array}\right. \end{array} $$

which leads to Eq. C2. In this equation, *e*_*k*− 1_ and *e*_*k*_ refer to the number of equality constrained parameters that are collapsed in *𝜃*_*k*− 1_ and *𝜃*_*k*_, respectively. In the example above, this means that to determine the lower bound for *𝜃*_∗_ we multiply the preceding value *𝜃*_1_ by three, such that the lower bound is $\left (\frac {0.05}{1}\right )\times 3 = 0.15$. In addition, to determine the lower bound of *𝜃*_5_ we divide the preceding value *𝜃*_∗_ by three, that is, $\left (\frac {0.45}{3}\right ) \times 1 = 0.15$. Similarly, to determine the upper bound for a given parameter value *𝜃*_*k*_, we need to multiple the upper bound by the number of parameters that are collapsed within it:

C4 $$ \begin{array}{@{}rcl@{}} u_{k} = \left\{\begin{array}{ll} \frac{1}{ERS} \times e_{k} & \text{if } k = 1 \\ \frac{1 - {\sum}_{i < k} \theta_{i}}{ERS} \times e_{k} & \text{if } 1 < k < K, \end{array}\right. \end{array} $$

where $1 - {\sum }_{i < k} \theta _{i}$ represents the length of the remaining stick and the number of elements in the remaining stick are computed as follows: $ERS = {{\sum }_{k}^{K}} e_{k}$. For the example above, the upper bound for *𝜃*_∗_ is $\frac {1 - 0.05}{5} \times 3 = 0.57$. The upper bound for *𝜃*_5_ is then $\frac {(1 - 0.05 - 0.45)}{2} \times 1 = 0.25$.

#### Corrections for free parameters

Different adjustments are required for a sequence of inequality constrained parameters that share upper and lower bounds. Consider, for instance, a multinomial model in which we specify the informed hypothesis

$$ \mathcal{H}_{r}: \theta_{1} < (\theta_{2}  ,  \theta_{3}) < \theta_{4}. $$

This hypothesis specifies that *𝜃*_2_ and *𝜃*_3_ have the shared lower bound *𝜃*_1_ and the shared upper bound *𝜃*_4_, however, *𝜃*_2_ can be larger than *𝜃*_3_ or vice versa. To integrate these cases within the stick-breaking approach one must account for these potential changes of order. For these cases, the lower bounds for the parameters remain unchanged. To determine the upper bound for *𝜃*_*k*_, we need to subtract from the length of the remaining stick the lower bound from the parameters that are free to vary. However, only those parameters are included in this calculation that have not yet been transformed:

C5 $$ \begin{array}{@{}rcl@{}} u_{k} = \left\{\begin{array}{ll} \frac{1 - (f_{k} \times l_{k})}{K} & \text{if } k = 1 \\ \frac{1 - {\sum}_{i < k} \theta_{i} - (f_{k} \times l_{k})}{ERS} & \text{if } 1 < k < K, \end{array}\right. \end{array} $$

where *f*_*k*_ represents the number of free parameters that share common bounds with *𝜃*_*k*_ and that have been not yet been transformed. Here, the number of elements in the remaining stick is defined as the number of all parameters that are larger than *𝜃*_*k*_: $ERS = 1 + {\sum }_{j > k} f_{j}$. To illustrate this correction, assume that samples from the encompassing distribution take the values (0.15, 0.29, 0.2, 0.36). The upper bound for *𝜃*_1_ is simply $\frac {1}{4}$. For *𝜃*_2_, we need to take into account that *𝜃*_2_ and *𝜃*_3_ share common bounds. To compute the upper bound for *𝜃*_2_, we subtract from the length of the remaining stick the lower bound of *𝜃*_3_: $\frac {1 - 0.15 - (1 \times 0.15)}{1 + 1} = 0.35$.

A further correction is required if a preceding free parameter (i.e., a parameter with common bounds that was transformed already) is larger than the upper bound of the current parameter. For instance, in our example the upper bound for *𝜃*_3_ would be $\frac {1 - 0.44 - 0}{1 + 1} = 0.28$, which is smaller than the value of the preceding free parameter, which was 0.29. If in this case *𝜃*_3_ would actually take on the value close to its upper bound, for instance *𝜃*_3_ = 0.275, then—due to the sum-to-one constraint—*𝜃*_4_ would violate the constraint (i.e., $0.15 < (0.29 , 0.275) \nless 0.285$). In these cases, the upper bound for the current *𝜃*_*k*_ needs to be corrected downwards. To do this, we subtract from the current upper bound the difference to the largest preceding free parameter. Thus, if $u_{k} < \max \limits (\theta _{i < k})$, the upper bound becomes:

C6 $$ \begin{array}{@{}rcl@{}} u_{k} &=& u_{k} - (\max(\theta_{i < k}) - u_{k}) \end{array} $$

C7 $$ \begin{array}{@{}rcl@{}} &=& 2 \times u_{k} - \max(\theta_{i < k}). \end{array} $$

For our example the corrected upper bound for *𝜃*_3_ would become 2 × 0.28 − 0.29 = 0.27 which secures the proper ordering for the remainder of the parameters. If in this case *𝜃*_3_ would take on the value close to its upper bound, for instance *𝜃*_3_ = 0.265, *𝜃*_4_—due to the sum-to-one constraint—would take on the value 0.295 which would be in accordance with the constraint (i.e., 0.15 < (0.29, 0.265) < 0.295).

## Footnotes

1 In addition, the function to compute the relative mean square error for bridge sampling estimates in **multibridge** is based on the code of the error_measures-function from the **bridgesampling** package.

2 Nuijten et al., (2016) did not report inferential tests because they had sampled the entire population. We do report inferential tests here because we wish to learn about the latent data-generating process.
